# Decoding internally generated transitions of conscious contents in the prefrontal cortex without subjective reports

**DOI:** 10.1038/s41467-022-28897-2

**Published:** 2022-03-22

**Authors:** Vishal Kapoor, Abhilash Dwarakanath, Shervin Safavi, Joachim Werner, Michel Besserve, Theofanis I. Panagiotaropoulos, Nikos K. Logothetis

**Affiliations:** 1 Department of Physiology of Cognitive Processes, Max Planck Institute for Biological Cybernetics, Tübingen, 72076 Germany; 2 International Center for Primate Brain Research, Center for Excellence in Brain Science and Intelligence Technology (CEBSIT), Institute of Neuroscience (ION), Chinese Academy of Sciences, Shanghai, China; 3 International Max Planck Research School, Tübingen, 72076 Germany; 4 Department of Empirical Inference, Max Planck Institute for Intelligent Systems, 72076 Tübingen, Germany; 5 Cognitive Neuroimaging Unit, CEA, DSV/I2BM, INSERM, Universite Paris-Sud, Universite Paris-Saclay, Neurospin Center, 91191 Gif/Yvette, France; 6 Division of Imaging Science and Biomedical Engineering, University of Manchester, Manchester, M13 9PT UK

**Keywords:** Consciousness, Sensory processing, Visual system

## Abstract

A major debate about the neural correlates of conscious perception concerns its cortical organization, namely, whether it includes the prefrontal cortex (PFC), which mediates executive functions, or it is constrained within posterior cortices. It has been suggested that PFC activity during paradigms investigating conscious perception is conflated with post-perceptual processes associated with reporting the contents of consciousness or feedforward signals originating from exogenous stimulus manipulations and relayed via posterior cortical areas. We addressed this debate by simultaneously probing neuronal populations in the rhesus macaque (Macaca mulatta) PFC during a no-report paradigm, capable of instigating internally generated transitions in conscious perception, without changes in visual stimulation. We find that feature-selective prefrontal neurons are modulated concomitantly with subjective perception and perceptual suppression of their preferred stimulus during both externally induced and internally generated changes in conscious perception. Importantly, this enables reliable single-trial, population decoding of conscious contents. Control experiments confirm significant decoding of stimulus contents, even when oculomotor responses, used for inferring perception, are suppressed. These findings suggest that internally generated changes in the contents of conscious visual perception are reliably reflected within the activity of prefrontal populations in the absence of volitional reports or changes in sensory input.

## Abstract (web summary)

The role of the prefrontal cortex in conscious perception is debated because of its involvement in task relevant behaviour, such as subjective perceptual reports. Here, the authors show that prefrontal activity in rhesus macaques correlates with subjective perception and the contents of consciousness can be decoded from prefrontal population activity even without reports.

## Introduction

One of the most elusive problems in science is to understand the biological basis of consciousness^1^. A seminal paper almost 30 years ago incited researchers that *“the time is ripe for an attack on the neural basis of consciousness”* and proposed conscious visual perception as a form of consciousness within the reach of neuroscience^2^. Since then, several theoretical treatises^3–5^, including the frontal lobe hypothesis^3^, higher order (HOT)^5^ and global neuronal workspace (GNW) theories^4,6^, postulate the prefrontal cortex (PFC) as a critical node in mediating conscious perception. Evidence supporting its involvement comes from functional magnetic resonance imaging (fMRI)^6–8^, experience of visual hallucinations upon its electrical stimulation^9,10^, impaired conscious processing following PFC lesions^11–15^ and intracortical recordings^16–20^.

Alternative approaches such as the integrated information (IIT) or the recurrent processing (RPT) theories diverge on the contribution of PFC to conscious perception^21,22^. Their proponents suggest that activity in posterior cortical areas reflects conscious contents, and PFC is primarily responsible for its executive role of processing the behavioral and cognitive consequences of conscious perception like task demands, introspection about the perceived stimuli and motor reports of perception^23–27^. This is corroborated with functional magnetic resonance imaging (fMRI) studies utilizing paradigms where either no volitional report is required or perceptual changes are indiscernible and therefore unreportable^23,26,28^. These studies suggest that consequences of perception such as decision making or motor action drive frontal activity, thus casting doubt if PFC represents conscious content^8,11,25,27^. However, the univariate contrastive fMRI analysis, as well as the indirect nature and limited spatial resolution of BOLD fMRI signal compared to direct neuronal recordings^29^ leaves open the possibility that prefrontal ensembles could reflect the content of consciousness even without report requirements or other conflating variables. Importantly, this cannot be firmly established from previous electrophysiological studies probing the PFC during conscious perception since they either leveraged an external manipulation of stimulus presentation, which affected the feedforward sensory drive^17^ or utilized motor reports^16,18–20^. These variables could conflate the signals intrinsically related to conscious perception (also see Supplementary Table 1). For example, it is possible that prefrontal activity during conscious perception paradigms utilizing external changes in sensory input reflects feedforward signals relayed via the posterior cortical areas^17^. In addition, if motor reports are required to indicate conscious perception, it is plausible that prefrontal signals could reflect the decision about the specific motor report that is associated with a particular visual stimulus^16,18–20^. Therefore, ascertaining the presence of conscious content related signals in the PFC, in the absence of these variables, is necessary for a resolution of the aforementioned debate and crucial for understanding whether PFC is a part of the cortical network underlying conscious perception.

We examined this by simultaneously probing the activity of neuronal populations in the macaque ventrolateral PFC with multi-electrode arrays during a no-report binocular rivalry (BR) paradigm. BR is a phenomenon, wherein presentation of incongruent, dichoptic visual input to corresponding retinal locations results in stochastic, endogenously driven alternations in subjective perception of the presented stimuli. It therefore allows a dissociation of conscious perception from sensory input, since the contents of consciousness fluctuate without a change in sensory stimulation^30,31^. Typical BR experiments require perceptual reports from subjects, thus conflating the neural activity related to consciousness with signals related to its consequences such as volitional reports, decision making and introspection^23,32–34^. Objective indicators of perception like the optokinetic nystagmus (OKN) reflex, a combination of smooth pursuit and fast saccadic eye movements, provide a solution to this problem. OKN polarity correlates with the perceptual reports during BR elicited with stimuli containing opposing motion content^35,36^. Thus, exploiting OKN as a surrogate measure to infer conscious perception can address confounds in the neural activity originating from motor reports. Importantly, utilizing such a stimulus set allows scrutinizing, if PFC represents simple low-level features such as direction of motion, when they are consciously perceived. To this end, we probed prefrontal ensembles in rhesus macaques (which were not trained for reporting their perception during a behavioral task) while they experienced no-report BR to investigate representations of conscious content in the PFC. Therefore, a central aim of the present work was to unravel the report-free prefrontal correlates of conscious visual perception during internally generated changes in the contents of consciousness.

Here we show that stimulus selective, prefrontal activity is modulated in concordance with subjective perception, during both externally induced and internally generated changes in conscious perception. Importantly, we reliably decoded the contents of conscious perception from prefrontal activity in the absence of subjective reports. These findings suggest a decodable representation of the ongoing conscious percept within an executive brain region such as the PFC.

## Results

### No-report BR paradigm and the temporal dynamics of perception

**Fig. 1:**
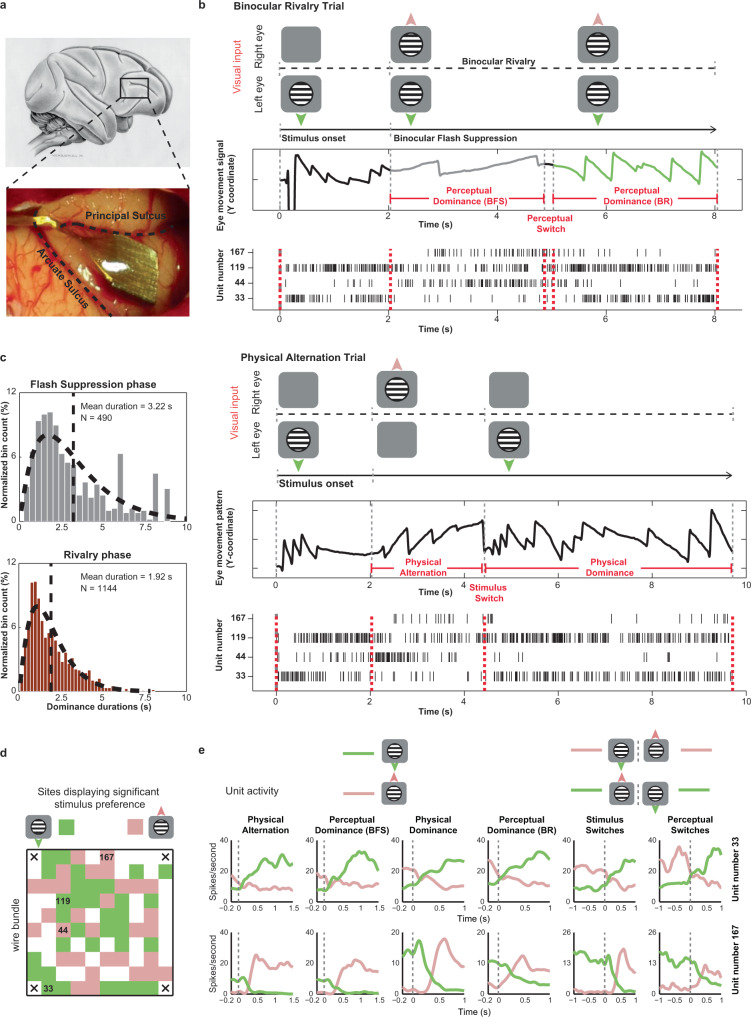
Recorded area, BR paradigm and example unit activity. **a**, Utah array location displayed over the vlPFC (schematic macaque brain and post implantation location in one animal). **b**, Visual input, OKN and spiking activity during an example BR and PA trial. A drifting sinusoidal grating (downward) was monocularly presented first during both trials. During BR, an upward drifting grating was presented to the contralateral eye 2000 ms later, resulting in perceptual suppression (BFS) of downward motion, as inferred from the OKN (grey curve). Externally induced perceptual suppression lasted ~3000 ms, following which a spontaneous switch (~5000 ms) reinstated the perception of downward motion (green curve). Units 33 and 119 display strong spiking activity when downward drifting grating is perceptually dominant, while units 44 and 167 respond more when upward drifting grating is perceived. Below is a PA trial. Following initial monocular presentation of downward drifting grating, an upward drifting grating was presented to the contralateral eye. The stimulus was switched later (~4500 ms) resulting in a change in OKN polarity. Individual units were preferentially modulated by similar direction of motion as during BR **c**, Perceptual dominance distributions during flash suppression and rivalry phases (derived from the OKN traces) are approximated well by a gamma distribution. **d**, Sites with significant stimulus preference (Wilcoxon rank-sum test, two-sided, *p* ≤  0.05) during PA trials (physical alternation phase) projected on the array for one recording session (selectivity here was computed using spiking activity recorded from a given electrode). Location of units (identified after spike sorting) presented in b are marked. Green and pink pixels reflect sites, where activity was stronger for downward or upward drifting gratings, respectively. **e**, Average spike density functions of two simultaneously recorded PFC units, 33 (preferred downward motion) and 167 (preferred upward motion), during PA and BR trials. Pink and green colors in the first four columns depict responses pertaining to downward and upward drifting grating respectively. The last two columns display the activity during a stimulus or perceptual switch from downward to an upward drifting grating (pink) and vice versa (green). Source data are provided as a Source Data file.

The no-report BR paradigm consisted of two trial types, physical alternation (PA, wherein perceptual changes were induced externally without visual competition) and binocular rivalry (BR, where perceptual changes could also occur endogenously because of visual competition, see Fig. 1b and Supplementary Fig. 1 and methods). Each trial started with a fixation spot cueing the animal to initiate fixation, lasting ~300 milliseconds, followed by monocular presentation of a stimulus (upward or downward drifting motion) for 1 or 2 sec. After this, an oppositely drifting stimulus (grating or random dots) was presented to the contralateral eye during BR trials, typically inducing perceptual suppression of the first stimulus, a phenomenon termed as binocular flash suppression (BFS)^37,38^. BFS, typically resulted in a switch of the OKN polarity (Fig. 1b), indicating perception of the newly presented direction of motion (Fig. 1b, marked in grey). Following this, visual competition ensued, resulting in spontaneous perceptual switches between the two stimuli as inferred from the OKN polarity. We evaluated the onset and offset of perceptual dominance periods during every trial based on the stability of the OKN pattern (see methods) during BFS and BR. Perceptual dominance durations displayed a gamma distribution (Fig. 1c), a key characteristic of multistable perception dynamics^39^, which has been previously established for both human and monkey observers that reported their perception^40^.

### Feature selective PFC activity during changes in conscious perception

We targeted the inferior convexity of the PFC (Fig. 1a and methods), where neurons display selective responses to complex visual stimuli and direction of motion^41–43^. Neural representations of conscious content are assessed with such feature selectivity. If neurons or units (see methods and see below) during BR reliably increase and decrease their firing rate contemporaneously with the perceptual dominance and suppression of their preferred stimulus respectively, then they are considered perceptually modulated and provide an explicit representation of conscious content^40^. We spike sorted and identified putative single units in our recordings. The spiking waveforms, recorded from a given electrode, which could not be sorted to a given single unit, were collected and denoted as a multi-unit. The valid spiking activity recorded from a given electrode together is referred to as a site. For a majority of analyses presented in this study (unless stated otherwise), we utilized the sorted single units and multi-units and they are collectively referred to as units. After sorting, we identified 987 units across two animals (734 units from H07, and 253 units from A11). We would like to add that the chronic nature of recordings might lead to an overlap of units across different recording sessions. At the same time, our approach is similar to other recent studies, wherein recording sessions (with chronically implanted arrays) on separate days are treated as independent^44–46^. We further note that we also carried out the decoding analysis (presented later) on individual recording sessions, which alleviates the aforementioned concern.

Figure 1b displays discharges of four such, simultaneously recorded prefrontal units and OKN during one PA and one BR trial. While two units fired more when the downward drifting grating was presented, another two displayed stronger modulation for the opposite motion direction during a PA trial (Fig. 1b). Spiking activity of these units was similarly modulated with subjective changes in conscious perception during a BR trial, for both externally induced perceptual suppression (BFS) and a subsequent spontaneous switch in conscious content (BR) (Fig. 1b).

We analyzed the spiking activity of the recorded units separately during perceptual dominance and suppression periods either (i) externally induced during BFS, or (ii) brought about by an endogenous spontaneous switch in BR. Furthermore, selectivity of neural activity was analyzed both before and after such perceptual switches and in all aforementioned cases, compared with selectivity in corresponding temporal phases during PA trials (see methods). We found sites displaying similar as well as opposite stimulus preference (as judged from their underlying spiking activity) distributed in close proximity throughout the electrode array (Fig. 1d and Supplementary Fig. 2a). Individual units recorded from these sites displayed robust perceptual modulation in their average response (examples in Fig. 1e and Supplementary Fig. 2).

### Strength of unit selectivity and population responses

**Fig. 2:**
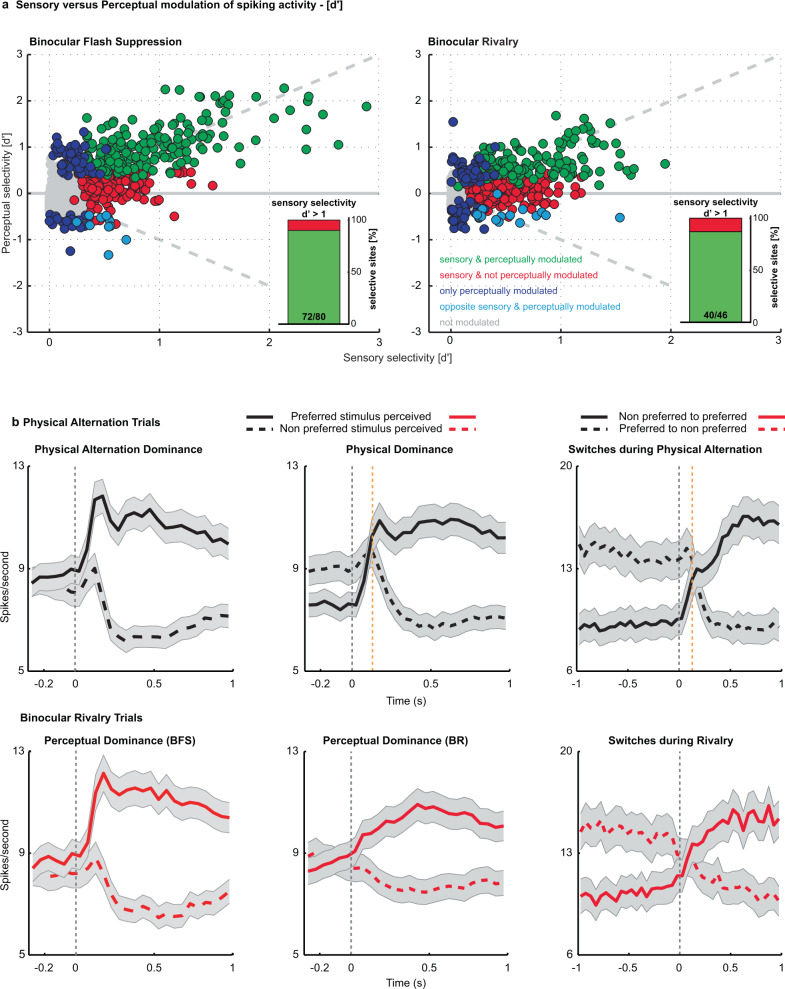
Sensory (PA) versus perceptual (BR) modulation of spiking activity - d’ and average population activity. **a**, Scatter plot of sensory vs. perceptual selectivity (d′) for all units (dots) during BFS and BR. Displayed with different colors are units; showing no significant modulation in PA or BR trials (grey); those with significant modulation for the same stimulus during both trial types (green); units displaying significant preference only during PA (red) and units displaying significant modulation only during BR trials (blue) and a small percentage of units which fired more when their preferred stimulus was perceptually suppressed (cyan). Proportion of perceptually modulated units for both BFS and BR increased as a function of sensory selectivity strength (insets showing perceptual modulation for d’>1, BFS - 90% and BR - 86%). **b**, Population activity averaged across units, which were significantly modulated during PA (upper row, presented in black) or BR (lower row, presented in red) trials and preferred the same stimulus, is plotted for perceptual dominance brought about by BFS (left) or during BR (middle), as well as switches (right) during BR. Plotted above is population activity during temporally matched phases in PA. Shaded regions depict standard error of the mean. The orange dashed line indicates the average delay between the physical stimulus transition and the OKN derived transition during PA trials (129.4 ± 36.6 ms following the onset of change in the physical stimulus). A remarkable similarity in population activity across trial types indicates robust perceptual modulation. Source data are provided as a Source Data file.

We estimated the selectivity of the recorded units (see methods) during sensory, monocular presentations in PA trials and compared it with their selectivity during subjective perception in BFS and BR (see methods). The majority of stimulus selective units (as judged from PA trials) fired on average more when their preferred stimulus was consciously perceived compared to its perceptual suppression (in BR trials). Specifically, during the BFS and BR phases of the paradigm, 84.21% (288/342) (H07: 87.72% (250/285); A11: 66.67% (38/57)) and 76.09% (277/364) (H07: 75.52% (216/286); A11: 78.21% (61/78)) of stimulus selective units, respectively displayed on average stronger activity during the perceptual dominance of their preferred stimulus (with 53.8% (184/342) (H07: 60.35% (172/285); A11: 21.05% (12/57)) and 40.38% (147/364) (H07: 42.31% (121/286); A11: 33.33% (26/78)) being significantly modulated, Wilcoxon rank-sum test, p  ≤ 0.05; also see Supplementary Table 2 and Fig. 2a). This result indicates that conscious content is robustly encoded in PFC. While many units fire more during perceptual suppression of their preferred stimulus in earlier visual regions (proposed as a part of an inhibitory mechanism independent of the mechanisms of perception)^30^; such units were a minority in PFC (BFS-2.92% (10/342) (H07: 2.81% (8/285); A11: 3.51% (2/57)) and BR-4.12% (15/364) (H07: 4.89% (14/286); A11: 1.28% (1/78))). Moreover, several units displayed significant preference only during BFS (26.51%, 70/264) (H07: 24.05% (57/237); A11: 48.15% (13/27)) and BR (34.41%, 85/247) (H07: 35.71% (75/210); A11: 27.03% (10/37)), indicating a separate population, whose activity discriminates across the subjective perception of the two visual stimuli reliably, only during visual competition. The pattern of results were largely comparable across animals.

In general, units displayed considerable heterogeneity in stimulus preference strength estimated using a d’ index (see methods) during the temporal phases of BFS (d’-physical alternation = 0.3985 ± 0.0131 (Mean±SEM), d’-perceptual dominance = 0.4471 ± 0.0133) and BR (d’-physical dominance = 0.3075 ± 0.0098, d’-perceptual dominance = 0.2719 ± 0.0082). The magnitude of sensory selectivity (d’) was a critical factor determining perceptual modulation (Fig. 2a) since almost 90% of units displaying strong sensory selectivity (d’ > 1), fired more during conscious perception of their preferred stimulus during BR trials (Wilcoxon rank-sum test, *p*  ≤ 0.05) (90% (72/80) for BFS and 86.96% (40/46) for BR). These proportions are remarkably similar to those observed in the temporal lobe of macaques participating in report based BR^47^, thus indicating that neuronal activity in two cortical regions reflects conscious content. Further, these results suggest that PFC activity correlates with externally and internally driven switches in subjective conscious perception of simple visual features like direction of motion, in addition to the externally induced perceptual suppression of faces and more complex stimuli^17^.

Next, we computed the prefrontal population spiking activity by averaging the mean activity of all units that displayed significant modulation (in PA or BR) and similar preference across temporally corresponding phases of PA and BR trials (see methods). Similar to their response during monocular presentations (PA trials), the average population response during both BFS and BR phases displayed stronger activity during conscious perception of a preferred stimulus and a dramatic reduction, when the same stimulus was perceptually suppressed (Fig. 2b, upper and lower row). Interestingly, the strong transient response observed during BFS after the externally induced perceptual dominance was absent in BR, after the spontaneously induced perceptual transitions. Since reliable perceptual modulation was detected in both BFS and BR during subjective perception, this feedforward response component is unlikely to be the main source of conscious perception representations in the PFC. However, we also note that the lack of a transient response during BR could possibly also result from a jitter of the perceptual changes relative to OKN changes across trials.

Moreover, neural activity switched reliably around both physical stimulus and perceptual switches (Fig. 2b). Similar results were obtained when the population activity was computed by averaging the responses of the units, selected based upon their significant modulation in either PA or BR trials (Supplementary Fig. 3). Additionally, when neural activity during PA was aligned to OKN changes (see methods), as was done for BR trials, we obtained similar results (Supplementary Fig. 4 and 5 and Supplementary Table 3). Together, these results indicate robust modulation of prefrontal spiking activity during conscious perception.

### Decoding the contents of consciousness from prefrontal ensembles

**Fig. 3:**
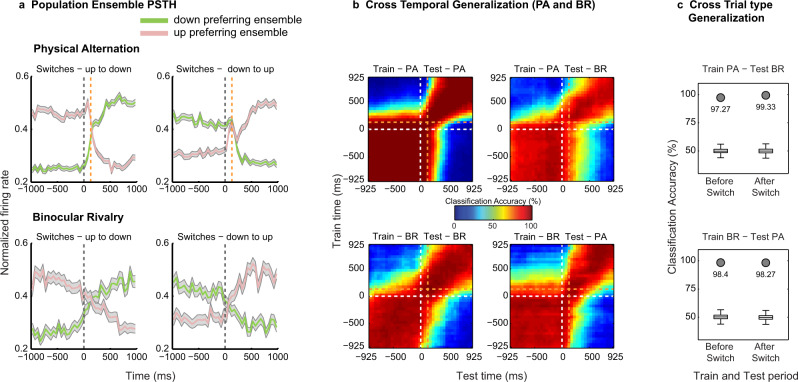
Decoding the contents of conscious perception from simultaneously recorded prefrontal ensembles. **a**, Normalized population spiking activity of down (green) and up (pink) preferring ensembles during up to down or down to up, PA (upper row) and BR (lower row) switches shows reliable modulation. Data are presented as mean, and shaded regions depict standard error of the mean. **b**, Cross-temporal decoding of conscious contents around switches during PA and BR trials and generalization across the two. Classification accuracy was computed for each pair of train and test time windows (see methods) in steps of 50 ms, using 150 ms bins. In both a and b, the delay between the physical stimulus transition and the OKN derived transition during PA trials is indicated with an orange dashed line (129.4 ± 36.6 ms). **c**, Cross trial-type invariance of the population code assessed by training a classifier on activity during one trial type and testing on the other, before and after a switch for a single 400 ms bin (starting 200 ms pre and post switch). Box plots (for box plot description, see statistical information, methods) depict the distribution of classification results with shuffled labels (*n* = 500), while filled circles denote the highly significant (permutation test, one-sided, estimated *p*-value: *p* = 0.00199) classification accuracy with real labels. Results suggest invariance of the population code, thus encoding perceptual contents. The presented results were computed with data from two animals pooled together. Similar results were observed for individual animals, which are presented in supplementary figure 6. Source data are provided as a Source Data file.

Probing the PFC with multi-electrode arrays allowed us to simultaneously monitor feature specific ensembles, that is, the two groups of units displaying preferential response to each motion direction. Ensemble activity reliably followed both exogenous stimulus changes during PA and endogenous perceptual transitions during BR trials (Fig. 3a). Importantly, this pattern of the population response during the two trial types was observed in prefrontal ensembles recorded in both animals (Supplementary Fig. 6a and d).

Next, we utilized a multivariate decoding approach^48^ to assess the population code’s ability to predict contents during single instances of stimulus and perceptual transitions (see methods). Classifiers trained on neural activity elicited around switches during PA or BR trials discriminated strongly above chance (50%) stimulus or perceptual contents not only within a trial type but crucially also generalized across them with near-perfect accuracy (levels reaching up to 95%, when computed over data collated across both animals, see Fig. 3b and c) revealing a relatively static population code^49^. Such temporal generalization was similarly observed in the population code, when the neural responses recorded in individual animals were subjected to the decoding analysis (Supplementary Fig. 6b, e). Importantly, strong temporal generalization of the classifier trained and tested before and after a switch, across PA and BR when the same stimulus was perceived, indicates an invariance in the population code representing sensory input and its subjective perception (Fig. 3c)^48^. Moreover, such an invariance is crucial to establishing that PFC represents the contents of conscious perception. For instance, if the PFC activity was reflecting the unconscious stimulus, the neural populations would be modulated differently during BR (in comparison to PA), when their preferred stimulus was perceptually suppressed. This would then result in a lack of generalization across the two trial types, PA and BR.

**Fig. 4:**
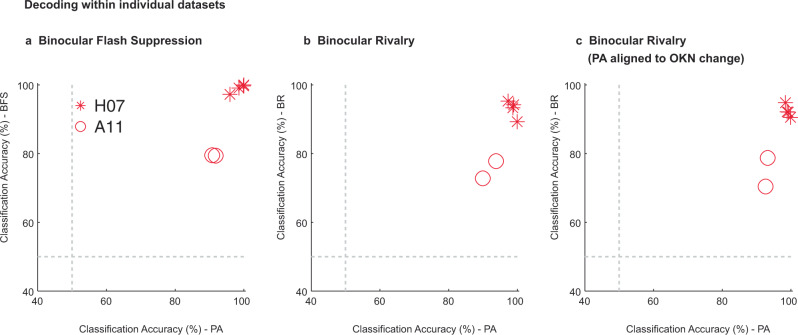
Assessment of decoding within individual datasets. This figure displays the classification accuracy at the level of individual recording sessions performed across the two animals: four sessions of H07 (red asterisks) and two sessions for A11 (red circles). Each point in the scatter plot corresponds to classification accuracy during PA or BR trials, obtained in a given dataset. The three panels present the results obtained across the different temporal phases of the paradigm: **a**, flash dominance, **b**, rivalry dominance, wherein PA trials are aligned to the physical stimulus change and **c**, rivalry dominance, wherein PA trials were aligned to the change in OKN. The results are computed over an 800 ms window starting 200 ms after the event used for aligning the data (physical stimulus change, or OKN derived change). We note that the classification accuracy for BR in **b** and **c** are similar but not identical, because they were obtained using two separate runs of the decoding analysis. In general, we observed strong classification accuracy even at the level of individual datasets.

This generalization across trial types during corresponding temporal phases was highly significant (permutation test, *p*  ≤ 0.002, see methods) when assessed during two temporal windows (400 ms), before (−200 ms to −600 ms) and after (200 ms to 600 ms) a switch (Fig. 3c). Similarly, reliable generalization across the two trial types was observed in both animals (Supplementary Fig. 6c and f). In contrast, training the classifier on activity before and testing after (or training on activity after and testing before) a switch resulted in a dramatic drop in the classification accuracy, given that oppositely drifting stimuli were perceived during the phases compared (Fig. 3b). Therefore, the population activity explicitly encodes the stimulus that enters awareness with minimal representation of the suppressed stimulus. Importantly, a similar overall pattern of results pertaining to the decoding analysis were obtained, when we analyzed the data from the two individual animals separately (Supplementary Fig. 6). Moreover, we observed robust decoding and generalization of stimulus and perceptual content within an individual dataset (see Supplementary Fig. 7 for results pertaining to one example dataset). Figure 4 summarizes the classification accuracy obtained within the two trial types for all individual datasets during the different temporal phases of the paradigm. Reliable classification and generalization accuracy was also obtained, when the PA activity was aligned to OKN change instead of stimulus changes (Supplementary Fig. 7). Together, these results suggest that the prefrontal population code underlying purely sensory perception without visual competition and subjective, conscious perception is similar, reliable and robust.

**Fig. 5:**
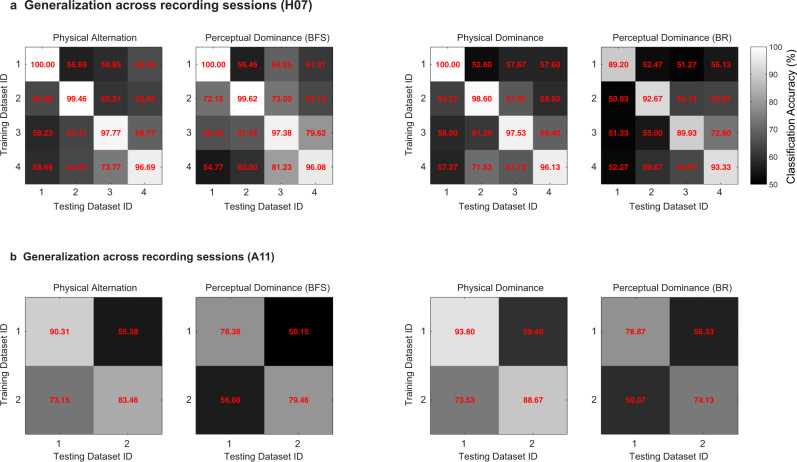
Decoding within and generalization across recording sessions. Displayed is the classification accuracy, **a**, computed for individual datasets and **b**, generalization accuracy across recording sessions. This analysis requires a correspondence of features across them. The units were identified after spike sorting, and can therefore be different across individual sessions. Thus, for this analysis, the spiking activity recorded from each of the 96 sites recorded with the array served as features, on which we trained and tested the classifier across the recording sessions. Results are presented for both the flash suppression (Perceptual dominance - BFS) and the rivalry phase (Perceptual dominance - BR) of the paradigm computed with neural activity elicited during an 800 ms window starting 200 ms after a stimulus or a perceptual change. The main diagonal (upper left to lower right) represents classification accuracy within a session and lateral diagonals represent generalization accuracy across sessions. While we find on average, high accuracy within individual recording sessions, the generalization across datasets is limited. Moreover, the classification accuracy within datasets was significantly more compared to generalization accuracy across datasets (two-sample *t-*test, two-sided, Physical Alternation_within_ vs. Physical Alternation_across_, *p* = 2.42*10^−9^, Perceptual Dominance (BFS)_within_ vs. Perceptual Dominance (BFS)_across_, *p* = 1.94*10^−5^, Physical Dominance_within_ vs. Physical Dominance_across_, *p* = 7.45*10^−10^, Perceptual Dominance (BR)_within_ vs. Perceptual Dominance (BR)_across_, *p* = 1.07*10^−7^). Potential reasons for such limited generalization could be that units either change or lose their preference, or it is likely, that we sampled from different units across recording sessions.

Further, we also assessed the ability of the population code to generalize across different recording days, by computing the generalization accuracy across recording sessions and compared it with the classification accuracy obtained within a recording session (see methods). Although strong decoding was observed within individual datasets (mean: Physical Alternation = 94.61, Perceptual Dominance (BFS) = 91.82, Physical Dominance = 95.78, Perceptual Dominance (BR) = 86.35), the generalization accuracy across recording sessions was limited (mean: Physical Alternation = 63.12, Perceptual Dominance (BFS) = 65.09, Physical Dominance = 62.57, Perceptual Dominance (BR) = 57.70) (see Fig. 5 and methods). This is in agreement with recent work^50^ which reported a steady turnover of individual neurons from one recording session to the next. This resulted in reduced prediction accuracy across sessions, when using fixed linear decoders, based directly on the recorded neural activity. Instead, it found low-dimensional latent dynamics were stable across days. The decreased generalization across recording days in our experiments, together with the strong classification performance within a day suggest that a similar turnover may have occurred in our recordings.

### Decoding motion content in the presence and during the suppression of eye movements

**Fig. 6:**
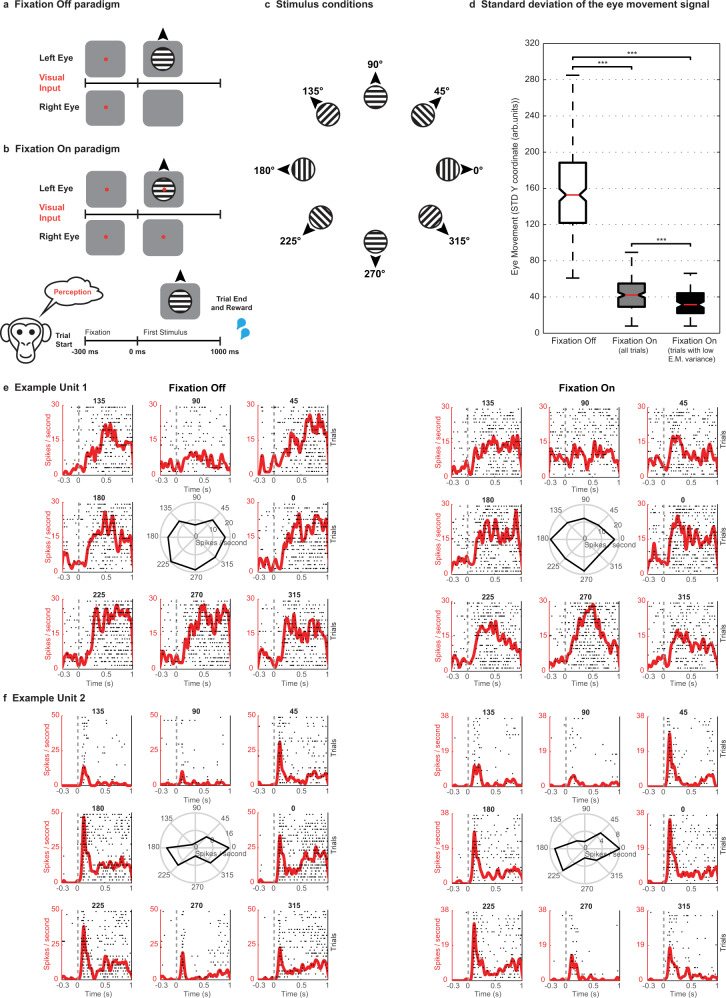
Control paradigms - Fixation Off, Fixation On and example unit activity. Trials started with a fixation spot, cueing the animal to bring and maintain gaze within a fixation window (300 ms), following which a drifting stimulus was presented monocularly. **a**, During Fixation Off, the fixation spot was removed at stimulus onset, thus inducing OKN. **b**, During Fixation On, the stimulus was presented without removal of the fixation spot, and the animal was required to maintain its gaze within a window (±1 or ±2°) until the trial ended, to receive a juice reward. **c**, During each trial, a stimulus drifting in one of eight different directions (pseudorandomized across trials) was presented. **d**, Whisker box plots (for box plot description, see statistical information, methods) displaying the distribution of standard deviations (STD) estimated from the eye movement signal (y-coordinate) elicited on individual trials during stimulus presentation (0–1000 ms). For Fixation On, either all (*n* = 187) or selected trials (*n* = 95), which displayed lower variance in the eye movement (E.M.) signal were analyzed (see methods). The STD was significantly reduced (Wilcoxon rank-sum test, two-sided, *** denotes *p*  ≤  0.001, Fixation Off vs. Fixation On (all trials), *p* = 7.86*10^−69^, Fixation On (all trials) vs. Fixation On (low E.M. variance), *p* = 9.07*10^−5^, Fixation Off (all trials) vs. Fixation On (low E.M. variance), *p* = 6.09*10^−46^) during Fixation On as compared to Fixation Off trials (*n* = 239). The results presented in this figure were computed with data from two animals pooled together. **e** and **f** show spike density functions overlaid on spike raster plots depicting the responses of two units to eight different motion directions during the two paradigms. The middle polar plots display the tuning curves of each unit (average response in Hz to gratings drifting in different directions). Spike rasters are displayed for first ‘*n*’ trials of every motion direction presentation. Here, *n* is the minimum number of trials presented to the animal across any motion direction during a given paradigm. PSTHs and tuning curves were computed taking all trials (of a given motion direction) into account. **e**, Example Unit 1 displays a stronger response to a stimulus drifting downwards during both paradigms. The unit displayed in **f** responds strongly to two opposite directions of motion, thus displaying orientation preference. Although the firing rate was higher during the Fixation off paradigm, the unit displayed similar preference across both paradigms. Source data are provided as a Source Data file.

**Fig. 7:**
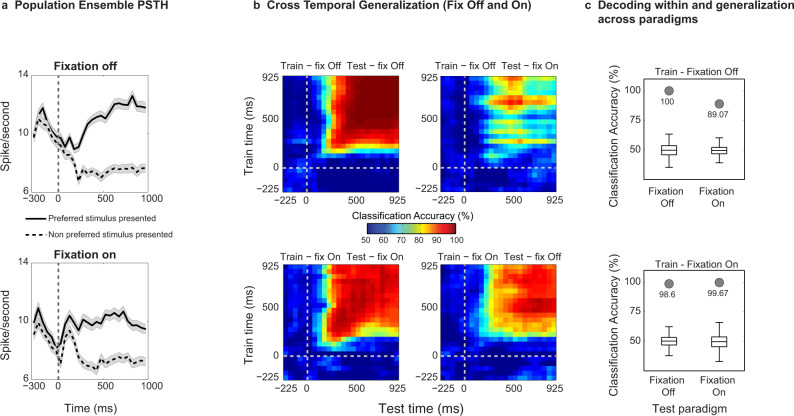
Decoding of motion content in the presence and during the suppression of OKN eye movements from simultaneously recorded prefrontal ensembles. **a**, Ensemble population spiking activity (see methods) during fixation-Off and fixation-On paradigm for units which were significantly modulated in either paradigms and preferred the same motion direction (also see methods). Black solid and dashed lines depict the response to the preferred and nonpreferred stimulus respectively. Data are presented as mean and shaded regions depict standard error of the mean. **b**, Cross-temporal decoding of stimulus contents (binning parameters similar to Fig. 3b). **c**, Cross-paradigm invariance of the population code was assessed by training a classifier on activity during one paradigm and testing on the other, for a single 400 ms bin (starting 400 ms poststimulus onset) during visual motion presentation. Significant (permutation test, one-sided, estimated *p*-value: *p* = 0.00199) accuracy (checked by comparing it to accuracy obtained with shuffled labels (*n* = 500), summarized with box plots (for box plot description, see statistical information, methods)) suggests that the population code is invariant to the presence of large OKN, and encodes stimulus motion contents. Classification accuracy for decoding within the paradigm is also presented. The presented results were computed with data from two animals pooled together. We observed similar pattern of results in individual animals, and they are presented in supplementary figure 8. Source data are provided as a Source Data file.

Finally, given the tight coupling between OKN and perceptual content, we aimed at dissociating neural activity related to oculomotor processes from activity related to visual input. For a majority of units (*n* = 581, H07 (*n* = 328), and A11 (*n* = 253)), we estimated their preference to direction of motion during two control experiments, which utilized the following two paradigms: Fixation Off and Fixation On. During Fixation Off, visual input elicited OKN, similar to the BR paradigm, while during Fixation On, macaques suppressed their eye movements by fixating a centrally presented spot (Fig. 6). Example units displaying similar tuning curves across the two paradigms are displayed in Fig. 6e and f. Our analysis focused on upward and downward motion, which were used for instigating competition during BR. Ensemble population PSTHs (see methods) of significantly modulated units during Fixation Off or Fixation On and preferring the same motion direction across the two conditions displayed stronger activity when the preferred motion direction was presented and a reduction in response to the nonpreferred visual input during both paradigms (Fig. 7a). A similar modulation of population responses was observed in both animals (Supplementary Fig. 8a and d). Further, a classifier trained on neural responses of these units to stimuli that elicited OKN could reliably predict the same stimuli with significant accuracy (permutation test, *p*  ≤ 0.002, see methods), when macaques viewed them with eye movements suppressed, and vice versa. Importantly, reliable generalization across paradigms was observed, when the classifiers were trained on the data collected from the two individual animals separately (Supplementary Fig. 8c and f). Therefore, this indicates that prefrontal activity contains stimulus information and is not just driven by eye movements (Fig. 7b and c). We also obtained similar results using only those Fixation On trials where eye movements within the fixation window were further controlled (see methods, Fig. 6 and Supplementary Fig. 9). Importantly the cross-paradigm generalization was robust to the unit selection procedure (see methods and Supplementary Fig. 10). These findings corroborate previous work suggesting that the frontal cortex responds to visual motion both in the presence and absence of OKN^43,51^ and indicate that motion content signals contribute to the activity of the tested population.

We further quantified the difference in the modulation strength of the unit population across the two control paradigms (Supplementary Fig. 11). This was assessed by computing a difference in the d-primes (fix Off – fix On) of individual units. Interestingly, the average of this difference was significantly different from zero for the last 500 ms (mean = 0.27 ± 0.07, one-sample *t-*test, *p* = 4.85*10^−4^), but not the first 500 ms (mean = −0.003 ± 0.05, one-sample *t*-test, *p* = 0.9552). A stronger response modulation appearing later in time during Fixation Off paradigm, is possibly related to a visual update, which is instigated by the saccade related to the optokinetic response. However, the similarity of this differential response (Supplementary Fig. 11a) during the first 500 ms across the two paradigms, suggests that prefrontal populations during this period represent direction of motion in the absence of any motor reports or when eye movements are suppressed. In summary, neurons in this prefrontal region reflect a mixture of perceptual and oculomotor signals^52^ and are selective for motion stimuli even when the monkeys fixate^43^. Oculomotor signals are therefore not solely responsible for prefrontal activity reflecting the contents of visual consciousness.

## Discussion

These results suggest that feature selective neural activity in the primate PFC reliably reflects internally generated changes in the content of subjective perception even without voluntary perceptual reports or external changes in visual stimulation, which would elicit strong feedforward responses. While addressing an ongoing debate among the various theories of consciousness regarding the neural correlates of conscious perception in the PFC^1,8,11,21,22,25,27,53^, we demonstrate that conscious contents can be reliably decoded from the activity of prefrontal ensembles.

A major debate in contemporary consciousness research focuses on the functional relevance of PFC in representing conscious perception. Multiple studies indicate a prefrontal involvement during paradigms investigating conscious perception^6–8,16–20,54^. However, recent theoretical and experimental work argue that previously reported frontal activation instead reflects prerequisites or consequences of perception^23–27^. In particular, it has been suggested that other cognitive processes mediated by the PFC such as decision-making, introspection or preparation and execution of motor reports (typical of these psychophysical paradigms), confound the previously observed correlates of conscious perception^23,32–34^. The opponents to a prefrontal role therefore posit that the correlates of conscious perception are limited to posterior sensory regions, which has instigated a ‘front vs back’ of the cortex debate regarding the cortical organization underlying conscious perception^1,11,27,54–57^. This is particularly relevant, since this critical issue separates key theories of consciousness with respect to their predictions about the role of PFC, in comparison to posterior cortices^1,55,58^. The present study addresses this debate by utilizing direct recordings of PFC neuronal populations during a no-report BR paradigm, wherein no volitional action was required from the subjects. In particular, this allowed us to carefully control for various cognitive processes associated with reporting, which may confound neural responses related to conscious perception. We find that prefrontal activity during BR reliably reflects changes in subjective perception, in the absence of these confounds.

BR offers a distinct advantage over other paradigms investigating visual consciousness such as BFS or visual masking, since it instigates stochastic, internally driven changes in the subjective perceptual content without a concomitant change in visual stimulation^30^. Hence, it confers an opportunity to observe the internal neural dynamics contemporaneous with spontaneous changes in the contents of visual consciousness, without the conflation introduced from abrupt changes in the sensory input affecting the feedforward drive, which is typical of other perceptual paradigms, such as binocular flash suppression^17,37^ (also see Supplementary Table 1). Paired with electrophysiological investigations of the primate visual system^59,60^, BR has revealed that the proportion of feature selective neurons reliably reflecting conscious content increases as one progresses in the visual cortical hierarchy from early visual areas to later temporal regions^40^. A recent study reported non-selective modulation of neural activity in human medial frontal cortex (in the anterior cingulate (ACC) and pre-supplementary motor area (pre-SMA)) before spontaneous perceptual transitions during BR, suggesting that some medial frontal areas might reflect the prerequisites of conscious perception^19^. In contrast, our results demonstrate conscious content-specific representations in a macaque ventrolateral PFC subregion, where cells are selective for faces, complex visual objects and direction of motion^41–43^ and which is reciprocally connected with the inferotemporal cortex^61^. Importantly, previous electrophysiological studies probing the PFC during conscious perception either utilized a motor report^16,18–20^ and were therefore likely conflated by consequences of conscious perception, or investigated perceptual modulation among neurons selective for high-level stimuli, such as faces and complex objects with a no-report BFS paradigm^17^. In BFS, perceptual dominance and suppression are externally induced due to an abrupt change in the visual stimulation that results in strong changes in the feedforward input (see Fig. 1 and Supplementary Table 1). In contrast, during BR, perceptual dominance and suppression are endogenously driven and therefore neural activity recorded during this paradigm is more likely to reflect conscious perception^17^, uncontaminated from the strong initial feedforward signal associated with a newly presented stimulus. Hence, our results collected during unreported but clearly occurring spontaneous transitions in conscious perception indicate the existence of prefrontal representations of conscious content. Together with results obtained from previous electrophysiological investigations employing BR, present results indicate that the neural correlates of conscious perception are distributed throughout the cortical hierarchy with stronger representations in association cortical areas, including the PFC.

These results constitute a critical advance in our understanding of PFC function. A quintessential node in the perception-action cycle, PFC has long been considered crucial for temporal organization of goal-directed behavior^62,63^. This is aided by its brain-wide anatomical connectivity, which includes both sensory and motor regions^64^. An important prerequisite suggested for implementing such executive function is a cognitively relevant representation of conscious perception in the PFC^63^. However, we never trained the animals participating in the current study to categorize or discriminate motion direction and report their perception, thus precluding an expectation that they were mentally generating an action, which was then withheld. Hence, our finding of report-free correlates of conscious content in the frontal cortex suggests that such perceptual information is available within the region, even when no rule-based overt action, such as a learned motor response associated with a specific stimulus, is required. Therefore, these results challenge the notion that post-perceptual processes, in particular, motor reports are the major source of PFC activity during paradigms of conscious perception^23,32^. They further challenge a purely task associated representation of perception in the PFC. Additionally, the diverse bi-directional connectivity of prefrontal cortex^64^ endows it with the ability to communicate this disambiguated ongoing perceptual content contemporaneously to multiple regions of the primate brain. This is particularly relevant in cases where top-down influences from the PFC might be required, such as cognitive control, attentional selection or visual processing^62,65^. Taken together, a holistic understanding of PFC function necessitates an investigation of areal and functional segregation of perceptual and cognitive processes within the region^66^.

In addition to finding conscious perception related signals in the PFC, we could reliably decode conscious content from prefrontal activity on individual trials with high accuracy. This is noteworthy, given that a multivariate pattern analysis approach, i.e., decoding, is considered a powerful framework for identifying the neural correlates of consciousness^67–69^, in comparison to univariate contrastive analysis. For example, decoding offers higher sensitivity since it uses the correlation or covariance of signals recorded across multiple units. Further, it confers an ability to judge the consistency with which the population code represents conscious content on a trial by trial basis, as well as across task conditions^68,69^. Moreover, it can provide insights into how neural representations transform in time by lending itself to a chronological evaluation of the population code^70^. In regards to this, our temporal generalization analysis revealed a population code, which was stable across time and robustly represented perception, within the PFC. This is likely important for mediating perceptual continuity^71^.

Crucially, we note that decoding of unambiguous stimuli (PA trials) alone cannot guarantee that neurons also reflect conscious perception of these stimuli. However, our result demonstrating the ability to decode with such high accuracy conscious perception from PFC activity during binocular rivalry, a visual stimulation protocol that dissociates conscious perception from visual input confirms decoding of subjective conscious perception, during visual competition. Specifically, the ability of the classifier to generalize across the unambiguous (PA) and ambiguous visual presentations (BR) suggests an invariance of the prefrontal population code underlying conscious content. In contrast, the absence of invariance between PA and BR could indicate representation of perceptually suppressed stimuli, therefore signaling the unconscious percept, whose representation seems to be minimal in PFC during BR. Interestingly, such a multiplexed representation of perceptually dominant and suppressed stimulus has been suggested recently in the case of temporal lobe neurons^72^.

Such a decoding approach could be used for investigating the population code subserving conscious perception across cortical regions, by for e.g., comparing decoding accuracy. However, the particularly strong classification and generalization accuracies (up to 95% obtained on data combined across both animals, see Fig. 3c) obtained from prefrontal responses, makes it unlikely that stronger decoding of the conscious percept would be observed from activity in other cortical areas.

Our findings are in contrast to the conclusions of recent imaging studies suggesting PFC’s reduced involvement in conscious perception^23,24,26^. However, constraints in the spatiotemporal resolution of the BOLD signal and its complex relationship with neural activity limit the interpretations from imaging data, especially so, when null findings are reported^29,54^. Such limits are particularly relevant to the frontal cortex, where individual neurons often display a high degree of mixed selectivity^73,74^ or distinct temporal patterns of activity during perceptual paradigms^66^. For example, we found that sites displaying preferential responses to opposite motion directions are distributed in close proximity (~0.4 mm) (Fig. 1d and Supplementary Fig. 2). Such spatial variability of stimulus selectivity remains difficult to capture with fMRI, especially with univariate methodologies. However, advances in high field imaging together with multivariate pattern analysis hold great promise in providing brain-wide representations of conscious content^67^.

In summary, our results lend support to theoretical approaches, which suggest a representation of conscious content in the executive areas of the brain such as the PFC to mediate cognitive functions or motor action^3,5,6,75^. Future work employing direct activation of such perceptually modulated ensembles could help elucidate the causal mechanisms mediating conscious perception^76^. Together with carefully designed experiments, it could help unravel the similarities and differences in the neural mechanisms underlying conscious perception and other cognitive processes^56^ such as introspection^23,77^, attention^78^, decision making^79,80^ or cognitive control^34,81^.

## Methods

All experiments were in full compliance with the guidelines of the European community (2010/63/EU) for the care and use of laboratory animals and were approved by the local authorities (Regierungspräsidium, Tuebingen, Baden-Württemberg, Germany, protocol KY6/12).

### Binocular rivalry, control paradigms, and stimulus presentation

The paradigm consisted of two trial types, namely, the physical alternation (PA) trials and binocular rivalry (BR) trials (Supplementary Fig. 1a). Both trial types started with the presentation of a red fixation spot (subtending 0.2 degree of visual angle), cueing the animal to initiate fixation. Upon successful fixation for 300 milliseconds within a fixation window (±8°), a drifting sinusoidal grating (size: 8°, speed: 12-13°/sec, spatial frequency: 0.5 cycles/degree, gratings were drifting vertically up or down) was monocularly presented. During one recording session (in A11), we used random dot motion stimulus (field of view 8°, speed 13°/sec, 200 limited lifetime dots, and 100% coherence) since visual inspection of OKN revealed that such a stimulus produced better OKN responses than drifting gratings in this animal. Further, it helped assess if prefrontal responses are correlated with perception of motion, independent of the low-level stimulus properties generating it. After one or two seconds, the first stimulus was removed and a second stimulus drifting in the opposite direction was presented to the contralateral eye in PA trials. During BR trials, the second stimulus was added to the contralateral eye without removing the first stimulus. This typically results in perceptual suppression of the first stimulus and is termed as flash suppression^17,37,38,82^ (Supplementary Fig. 1a). After this period, visual input alternated between oppositely drifting stimuli presented monocularly in the PA condition. In comparison, during the BR condition, the percept of the animal switched endogenously between the discordant visual stimuli. The temporal histogram of perceptual dominance during BR could be approximated with a gamma distribution (Fig. 1c). The total duration of a single-trial/observation period was around 8 to 10 s. Note that the perception of the animal (Supplementary Fig. 1a) is similar during the two trial types, even though the underlying visual input is monocular in PA trials, while it is dichoptic during BR. The eye (where the first stimulus was presented), motion direction (which was presented first), and trial types (PA or BR) were pseudorandomized within a single dataset. During the entire period of a trial, animals had been trained to maintain their gaze within a fixation window (±8°), which encompassed the stimulus. A liquid reward was given to the animal upon successful maintenance of gaze within the window for the entire trial duration.

The eye movement control experiments using the Fixation Off and Fixation On paradigms were carried out on a subset of recording sessions (4 out of 6 recording sessions, 2 - macaque H’07, 2 - macaque A’11). Both paradigms consisted of trials, where the macaques were presented with a visual stimulus drifting in one of eight different directions (pseudorandomized across trials) for one second (Fig. 6). Each trial started with the presentation of a fixation spot for ~300 milliseconds, following which a drifting visual stimulus was presented for one second. However, there was one key difference across the two paradigms. During Fixation Off, the fixation spot disappeared as soon as the visual stimulus was presented, eliciting OKN and the fixation window (the window within which the animal was required to maintain its gaze) encompassed the stimulus (±8°). In contrast, during the Fixation On paradigm, a fixation spot overlaid on the stimulus indicated that the monkeys must fixate within a smaller fixation window (~±1 to ±2 degrees) to complete the trial and receive the reward, thus suppressing eye movements. Stimulus parameters were identical to the ones used during the BR paradigm.

Dichoptic visual stimulation was carried out with the aid of a stereoscope and displayed at a resolution of 1280×1024 on the monitors (running at a 60 Hz refresh rate) using a dedicated graphics workstation. The custom-made stereoscope included two mirrors, slanted to approximately a 45° angle and placed in front of the animal’s eyes. The angle of the mirrors could be adjusted to allow precise binocular stimulation. In addition, the setup included two monitors, each on the left and right side, facing each other and perpendicular to the direction in which the animal faced. This allowed binocular stimulation, wherein images displayed on the left and right monitor could be presented to corresponding retinal locations of the left and right eye respectively, with the aid of slanted mirrors. Prior to the presentation of the BR paradigm, we carried out a previously described calibration procedure^82^, which ensured that the stimuli presented on the two monitors through the stereoscope were appropriately aligned and could be fused binocularly. It started with the animal participating in a fixation-saccade task, wherein visual input was at first presented monocularly to the left eye. The task required a brief period of fixation on a centrally presented red fixation spot (its location was adjusted according to single eye vergence for each individual monkey), following which a peripheral fixation target was presented in one of eight different directions. The animal was trained to make a saccade to the eccentrically presented target for obtaining a liquid reward. During this period, the eye position was centered within a fixation window, using a custom-designed linear offset amplifier. After this, a second procedure was carried out, wherein the fixation target was first presented to the left eye for a brief duration, after which it was switched off and immediately presented to the right eye. The animal typically responded with a saccade, whose amplitude provided an estimate of the offset between the fixation spot displayed on the two monitors. This offset was confirmed with several repetitions of this procedure and it served as a correction factor. The visual stimuli were aligned taking into account this correction factor, thus enabling their fusion.

Visual stimulus design and presentation were carried out using openGL (version 1.2) and controlled on a windows machine via Tcl/Tk (version 8.0). A QNX real-time operating system (QNX Software Systems, version 4.25, BlackBerry Limited, Waterloo, Canada) managed the precise temporal presentation of the visual stimuli, and sent digital pulses to the Blackrock recording system. An infrared camera captured eye movements (1 kHz sampling rate) with the software iView (SensoriMotoric Instruments GmBH, Germany). Besides monitoring eye movements online, they were also stored for offline analysis in both, the QNX-based acquisition system as well as the Blackrock neural data acquisition system. We used the latter to align the neural data.

### Surgical procedures

Two healthy male rhesus monkeys (*Macaca mulatta*), H07 and A11 participated in behavioral and electrophysiological recordings. H07 was ~12 years old and weighed ~11–12 kg at the time of the study. A11 was ~15 years old, and weighed ~8–9 kg, when the experiments were done. All experiments were approved by the local authorities (Regierungspräsidium, protocol KY6/12 granted to TIP as the principal investigator) and were in full compliance with the guidelines of the European community (2010/63/EU) for the care and use of laboratory animals. Each animal was implanted with a titanium headpost custom designed to fit the skull based on a high-resolution MR scan collected using a 4.7 tesla scanner (Biospec 47/70c; Bruker Medical, Ettlingen, Germany). The headpost implantation was carried out while the animal was under general anesthesia and prior to the beginning of behavioral training in the BR paradigm. Details of the surgical procedures have been previously described^83^. The MR scan also aided in localizing the inferior convexity of the lateral PFC. Post behavioral training in the task, the animals underwent another surgery, where a Utah microelectrode array (Blackrock Microsystems, Salt Lake City, Utah USA^84^;) was implanted in the PFC. The array had a 10 by 10 electrode configuration and was 4 mm by 4 mm in size, with an inter-electrode distance of 400 μm and electrode length of 1 mm. We implanted the array ventral to the principal and anterior to the arcuate sulcus, thus aiming to cover a large part of the inferior convexity in the ventrolateral PFC (Fig. 1a).

### Electrophysiology data acquisition

All behavioral training and electrophysiological recordings were carried out with the animals seated in a custom-designed chair. Data presented here was collected across six recording sessions in two macaques (4 - H’07 and 2 - A’11). Broadband neural signals (sampled at 30 kHz) were recorded with the Neural Signal Processors (Blackrock Microsystems) and band-pass filtered offline between 0.6 - 3 kHz using a second-order Butterworth filter. Spikes were detected with an amplitude threshold set at five times the median absolute deviation^85^. Any spike events larger than 50 times the mean absolute deviation were discarded. Further, spike events with an inter-spike interval of less than the refractory period of 0.5 ms were also discarded. Events satisfying the aforementioned criterion of threshold and the refractory period were kept for further analysis. Collected spike events were aligned to their minima and 45 samples (1.5 ms) around the peak were extracted for spike sorting. An automatic clustering procedure identified putative single neurons via a Split and Merge Expectation-Maximisation algorithm which fits a mixture of Gaussians on the spike feature data consisting of the first three principal components of the spike waveforms^86^. Inspection and manual cluster cutting was carried out in Klusters (Lynn Hazan, Buzsáki lab, Rutgers, Newark, United States)^87^. This way, we sorted and identified putative single units recorded from each of the 96 electrodes in the array. The spiking waveforms, recorded under a given channel, which could not be sorted to a given single unit were collected and denoted as a multi-unit. For the analysis presented in this study (unless stated otherwise), we combined individual single units and multi-units recorded and they are together referred to as units. After sorting, we identified a total of 734 units from H07 across four recording sessions, and 253 units from A11 across two recording sessions.

### Selectivity of unit activity

Eye movements during each BR trial were visually inspected with the aid of a custom written GUI in MATLAB (MathWorks, Natick, United States) and the onset and end of a perceptual dominance (during the rivalry phase) were manually marked using the onset of a change in the slow phase of the OKN as a criterion. Two authors VK and AD marked the datasets.

Selectivity of a given unit was assessed separately for PA and BR trials by comparing the spike counts elicited during the presentation (PA) or perception (BR) of downward vs. upward drifting stimuli, using a Wilcoxon rank-sum test (*p*  ≤ 0.05). For unit selectivity during BR trials, spiking response was aligned to the onset of two events, invoking a perceptual change, namely the (i) onset of flash suppression phase and (ii) onset of a period of perceptual dominance during spontaneous switches in rivalry. Unit selectivity was similarly assessed during analogous temporal phases of PA trials. The presentation of the second stimulus during PA is temporally corresponding with the presentation of the second stimulus during a BR trial, which constitutes the flash suppression phase. All subsequent stimulus presentations during a PA trial can be considered equivalent to the perceptual dominance phases during BR. Therefore, selectivity of the spiking responses during these periods was computed for assessing unit selectivity during PA trials. Further, we considered only those epochs during PA and BR trials for computing selectivity, which consisted of perceptual dominance (BFS and BR) or monocular presentation (PA) of a given stimulus lasting at least 1000 ms. Therefore, only those epochs of flash suppression dominance were included in the analysis, which entailed a successful suppression of the first monocularly presented stimulus for at least 1000 ms (as determined by the OKN). With respect to perceptual switches, we analyzed transitions, which consisted of at least 1000 ms of clear dominance (judged by a stable OKN pattern), before and after an OKN switch. To compare with PA as closely as possible, we analyzed those transitions during BR, which had an interval of less than 250 ms between the end of the preceding dominance, and the onset of the next. Data were aligned to the onset of the forward dominance. Corresponding temporal phases of stimulus switches during PA trials, included at least one second monocular presentation of a given stimulus followed by the presentation of an oppositely drifting stimulus in the contralateral eye (compared to the preceding visual presentation) for a minimum duration of 1000 ms. Selectivity was assessed both before (−1000 to 0) and after (0 to 1000) the perceptual (BR) and stimulus switches (PA) by collecting all spikes elicited in a 1000 ms period. Any relevant figures presented in the main body of the paper were obtained by analyzing the spiking activity elicited during PA trials which was aligned to the TTL pulse signaling a stimulus change. In addition, we visually inspected and marked the onset and offset of the visual stimulus during PA trials similarly to the way these episodes were marked for BR trials, based upon the change in the OKN direction. The selectivity analysis (Fig. 2) was repeated with PA trials aligned according to this new criterion and we obtained very similar results (Supplementary Fig. 4 and 5).

### D-prime calculation

For every unit, we computed a preference index denoted as d’, by quantifying the strength of its selectivity during PA trials. It was calculated as follows:

1 $${d}^{{\prime} }=(\mu p-\mu {np})/\left(\sqrt{\left(\frac{{{{{{\rm{Varp}}}}}}+{{{{{\rm{Varnp}}}}}}}{2}\right)}\right)$$

where, μp and μnp is the average spiking response of a given unit during the presentation of its preferred and nonpreferred stimulus, calculated over a duration of 1000 ms after a physical stimulus change during PA trials. The difference between these two quantities is normalized by the square root of the average pooled variance (Varp and Varnp) of the response distributions. The d’ was similarly calculated during corresponding periods of BR trials. However, the preferred and nonpreferred stimulus conditions were designated based on PA trials. A unit with a positive d’ during both PA and BR trials, signifies similar preference, while a negative d’ during BR indicates that the unit had opposite or no preference during PA (that is, it fired on average equal number of spikes for both motion conditions) (Fig. 2a and Supplementary Fig. 4a).

### Conventional population PSTHs and ensemble PSTHs

Population PSTHs (Fig. 2b) were computed by averaging the mean neural activity of selective units in response to their preferred and nonpreferred stimuli. The activity of each unit was calculated as the mean response of the unit during specific temporal phases (flash suppression, perceptual dominance and switches) in 50 ms bins during PA or BR trials. For the flash suppression and perceptual dominance phases, we identified all units, which displayed significant modulation during either PA or BR trials. With respect to switches, all units which displayed significant modulation (and maintained stimulus preference) before and after a switch during both trial types were identified. In all three cases, the population PSTH was computed by averaging the activity of all units, which displayed preference to the same motion direction across PA and BR. In addition, population PSTHs with units significantly selective in the PA or BR trials (Supplementary Fig. 3 and 5) were also computed. Population activity computed for the switches included units, which were significantly modulated both before and after the switch for the same motion direction in PA or BR (Supplementary Fig. 3 and 5).

Additionally, we generated average ensemble population PSTHs. Here we refer to a population of units displaying preference for the same stimulus as a neuronal ensemble. Unit populations, which contributed to ensemble PSTHs, were identified similarly to conventional population PSTHs. Therefore, the population of units contributing to Fig. 2b (switches) and Fig. 3a is identical. However, PSTHs were computed differently. First, the activity elicited by all units preferring the downward and upward motion directions were separately averaged for each transition in 50 ms bins, providing a population vector of each neural ensemble for every switch. Next, each of these traces was normalized by subtracting the minimum and dividing it by the maximum activity. Finally, traces were collected across all transitions across datasets and were averaged to generate the average ensemble population PSTHs, presented in Fig. 3a. Such an ensemble population PSTH complements the decoding approach, which utilizes the population response on single trials aimed at ascertaining the ongoing sensory input (PA) or perceptual experience (BR). Ensemble population PSTHs for the control paradigms presented in Fig. 7a, Supplementary Fig. 8a, d and 9a were generated similarly as described above, but without the normalization step. Further, the response of the two ensembles has been pooled together in this case. For the ensemble activity related to presentation of the preferred stimulus, all trials where the preferred stimulus of the units comprising the two different ensembles (upward and downward motion) were presented were pooled together and an average was computed. Similarly, all trials, where ensemble’s nonpreferred stimulus was presented, were pooled together and averaged for ensemble activity pertaining to the nonpreferred stimulus.

### Decoding Analysis

Multivariate pattern analysis was utilized to assess, if the spiking activity of neuronal ensembles in the PFC contained information about the contents of perception on a single transition basis. In this regard, we used a maximum correlation coefficient classifier^49^ implemented as a part of the neural decoding toolbox^88^. All the recorded units (*n* = 987) across the two monkeys were pooled as a pseudopopulation for the decoding analysis pertaining to the BR paradigm (Fig. 3). This is similar to previous studies^49,89^, where units recorded during independent sessions were compiled together as a pseudopopulation. For testing generalization across sessions (Fig. 5), each of the 96 individual electrodes of the array was considered a unit to maintain correspondence across sessions. The valid spiking activity recorded from this electrode was used for training and testing the classifier across the recording sessions. In either case (independent of how the units were defined), responses of each of these units during randomly selected stimulus (PA trials) and perceptual switches (BR trials) were utilized. A z-score normalization (subtracting the mean activity and dividing by the standard deviation) of each unit’s response was done before it participated in the classification procedure in order to assure that units with high spike rates do not influence the decoding procedure disproportionately. We used 15 cross-validation splits, implying that for 14 switches used for training the pattern classifier, one was left out and put in the test. For decoding during flash suppression dominance (presented in Fig. 4 and Fig. 5), the number of cross-validation splits were 13. This procedure was repeated 50 times (resample runs) to estimate the classification accuracy with a different randomly chosen cross-validation split during each run. All decoding accuracy estimates are zero-one-loss results. Each pixel in cross-temporal generalization plots (Figs. 3b, 7b and Supplementary Fig. 6b, e, 7b, e, 8b, e, 9b) depicts the classification accuracy computed with firing rates in 150 ms bins, sampled every 50 ms. This bin duration was chosen, because it has been previously used successfully for decoding visual input from neural activity recorded in the frontal and temporal cortex^49,89^.

Similar steps as described above were used for decoding analysis pertaining to the control paradigms (Fig. 7 and Supplementary Fig. 8 and 9), except the following. Units which were significantly selective (Wilcoxon rank-sum test, *p* ≤ 0.05) in either of the two paradigms and preferred the same stimulus, that is stronger response to the same motion direction during both paradigms (Final selection of units used: n = 103 (H07: *n* = 66, A11: *n* = 37) for Fig. 7 and *n* = 100 (H07: *n* = 65, A11: *n* = 35) for Supplementary Fig. 9) participated in the decoding procedure. Further, we employed eight cross-validation splits for the decoding analysis pertaining to trials with reduced eye movement variance (Supplementary Fig. 9).

Additionally, we wanted to assess the robustness of the cross-paradigm generalization across the control paradigms to the above mentioned unit selection procedure. To this end, we ran the decoding procedure ten times, and during each run, the units participating in the analysis were selected in the following manner. Similar to as described above, the units were chosen based on their selectivity (Wilcoxon rank-sum test, *p*  ≤ 0.05) in either of the two paradigms and similar preference (upward or downward) across them. However, this was computed differently: using all of the trials of one paradigm, which was used for subsequent training of the classifier, and only half of the trials of the second paradigm, which were randomly selected during each run. The generalization accuracy of the classifier was subsequently tested by training the classifier (on the activity of the set of units passing the aforementioned criterion) on the first paradigm and testing it on the remaining half of the trials from the second paradigm, which did not participate in the aforementioned unit selection procedure. The number of cross-validation splits employed for this analysis was seven. The rest of the decoding parameters were similar to as described above. We repeated this procedure 10 times, for both comparisons, that is, training the classifier on trials from Fixation Off and testing it on Fixation On, and vice versa (Supplementary Fig. 10). The resulting classification accuracies were strongly significant (*p*  ≤ 0.002), based on the permutation test.

### Selection of trials with reduced eye position variance and decoding

To create a robust dataset for decoding the visual stimulus during passive observation of monocularly presented stimuli, we only picked those trials of the Fixation On paradigm, wherein the eye movements during passive fixation were relatively minimal, that is, there were no strong drifts in eye position. The trial selection was carried out by utilizing the following procedure. Firstly, the Y coordinate of the eye movement signal was detrended. It was then filtered below 20 Hz to remove high-frequency noise. Next, a double differential of this signal was computed and compared to a flat line (i.e. with a slope of 0 and an intercept corresponding to the baseline of the OKN signal) using a least-squared-error minimization method. The sum of the squared error for each trial was computed giving us a distribution of errors. All trials within a condition, whose sum of least-squared error was less than the median of the distribution of these errors obtained from all trials in that condition, were selected for further analysis. This method resulted in a selection of trials with significantly (Wilcoxon rank-sum test, *p*  ≤  0.001) reduced variance of the eye movements signal (Fig. 6d).

### Statistical information

Significant selectivity of units was analyzed with a Wilcoxon rank-sum test during both PA and BR trials. We compared the spike responses elicited during specific temporal phases, namely the physical alternation, physical dominance, perceptual dominance during BFS or BR, as well as stimulus and perceptual switches as described above in the selectivity of unit activity section. The alpha value was 0.05.

Statistical significance of the classification accuracy was assessed using a permutation test, which involved running the decoding analysis on the data with labels shuffled^48,88^. This procedure was repeated 500 times with parameters related to binning, cross-validation splits as well as resample runs identical to those used for standard decoding of correctly labeled data. The resulting classification accuracies obtained served as a null distribution. If the decoding results obtained without shuffling the labels were greater than all values within the null distribution, they were considered as significant (*p*  ≤ 1/500 = 0.002, or an estimated *p*-value: *p* = 0.00199). Significance of decoding accuracy was computed using this procedure for the results presented in Figs. 3 and 7 (also see Supplementary Fig. 6, 7, 8, 9 and 10).

With respect to the box plots presented in Figs. 3c, 6d, 7c and Supplementary Fig. 6, 7, 8, 9 and 10, the box denotes the 25th (Q1) and 75th percentiles (Q3) of the data, while the red (Fig. 6d) or black central line (other figures) denotes the median. All adjacent values within Q3 + 1.5×(Q3−Q1) and Q1−1.5×(Q3−Q1) are contained within the upper and lower whisker lengths, respectively. Outliers are not displayed. In Fig. 6d, the 95% confidence interval around the median is approximated by the notches, whose edges are calculated as median ± 1.57×(Q3−Q1)/(square root of number of samples).

### Unit related statistics

The average spike density functions of two simultaneously recorded units, 33 (preferring downward motion) and 167 (preferring upward motion), which displayed robust modulation during both PA and BR trials are presented in Fig. 1e. These were plotted with the path function (which utilized a gaussian kernel with a standard deviation of 50 ms) from the Chronux data analysis toolbox (http://chronux.org/)^90^. The spiking activity of the units reliably switched for both externally induced and internally driven perceptual switches as assessed with a Wilcoxon rank-sum test. With respect to unit 33, the statistics of significant modulation were as follows: during physical alternation phase of PA trials, (temporally analogous to flash suppression dominance during BR), *p*_PA-33_ = 2.82*10^−14^ and for binocular flash suppression phase during BR trials, *p*_BFS-33_ = 2.39*10^−6^. Additionally, it was also significantly modulated during the physical dominance (PD) phase of PA trials and its temporally equivalent rivalry dominance (RD) phase during BR trials. The statistical value was *p*_PD-33_ = 8.72*10^−15^ for physical dominance and *p*_RD-33_ = 7.18*10^−8^ for rivalry dominance. For unit 167, the corresponding values were: *p*_PA-167_ = 1.13*10^−17^, *p*_BFS-167_ = 1.20*10^−9^, *p*_PD-167_ = 1.49*10^−4^ and *p*_RD-167_ = 7.8*10^−3^.

During the eye movement control experiments, a majority of units displaying significant stimulus selectivity (Wilcoxon rank sum test, p  ≤ 0.05) within a given paradigm retained their stimulus preference across the two control paradigms (fix On—69.56 %, 48/69 (H07: 70.83%, 34/48; A11: 66.67%, 14/21); fix Off—55.56 %, 80/144 (H07: 51.55%, 50/97; A11: 63.83%, 30/47)) while a small percentage of units (fix On—14.49 %, 10/69 (H07: 16.67%, 8/48; A11: 9.52%, 2/21); fix Off—6.94 %, 10/144 (H07: 8.25%, 8/97; A11: 4.26%, 2/47); Wilcoxon rank sum test, *p* ≤ 0.05) exhibited a significant preference to stimuli with opposing motion content across the two paradigms (see Fig. 6 for tuning curves of example units).

### Reporting summary

Further information on research design is available in the Nature Research Reporting Summary linked to this article.

## Supplementary information

Supplementary Information

Reporting Summary

## Data Availability

Source data are provided with this paper. Data with spiking activity, that support the findings of this study are available from the corresponding author upon reasonable request. Source data are provided with this paper.

## Source data

Source Data

## Code availability

We used codes, implemented as part of the neural decoding toolbox for carrying out the various decoding analysis reported within the paper. The toolbox (including the associated codes) is available for download at http://www.readout.info/downloads/. The utility of this toolbox has been previously described^87^. Other code is available from the corresponding author upon reasonable request.
