# Supplementary material for: Decoding internally generated transitions of conscious contents in the prefrontal cortex without subjective reports
Source: Nat Commun. 2022 Mar 22;13:1535. doi: 10.1038/s41467-022-28897-2 (PMC8940963; doi:10.1038/s41467-022-28897-2)
Supplement: Supplementary file 1 — Supplementary Information [file 41467_2022_28897_MOESM1_ESM.pdf]

SUPPLEMENTARY INFORMATION

Decoding internally generated transitions of conscious contents in the prefrontal cortex without subjective reports

SUPPLEMENTARY FIGURES

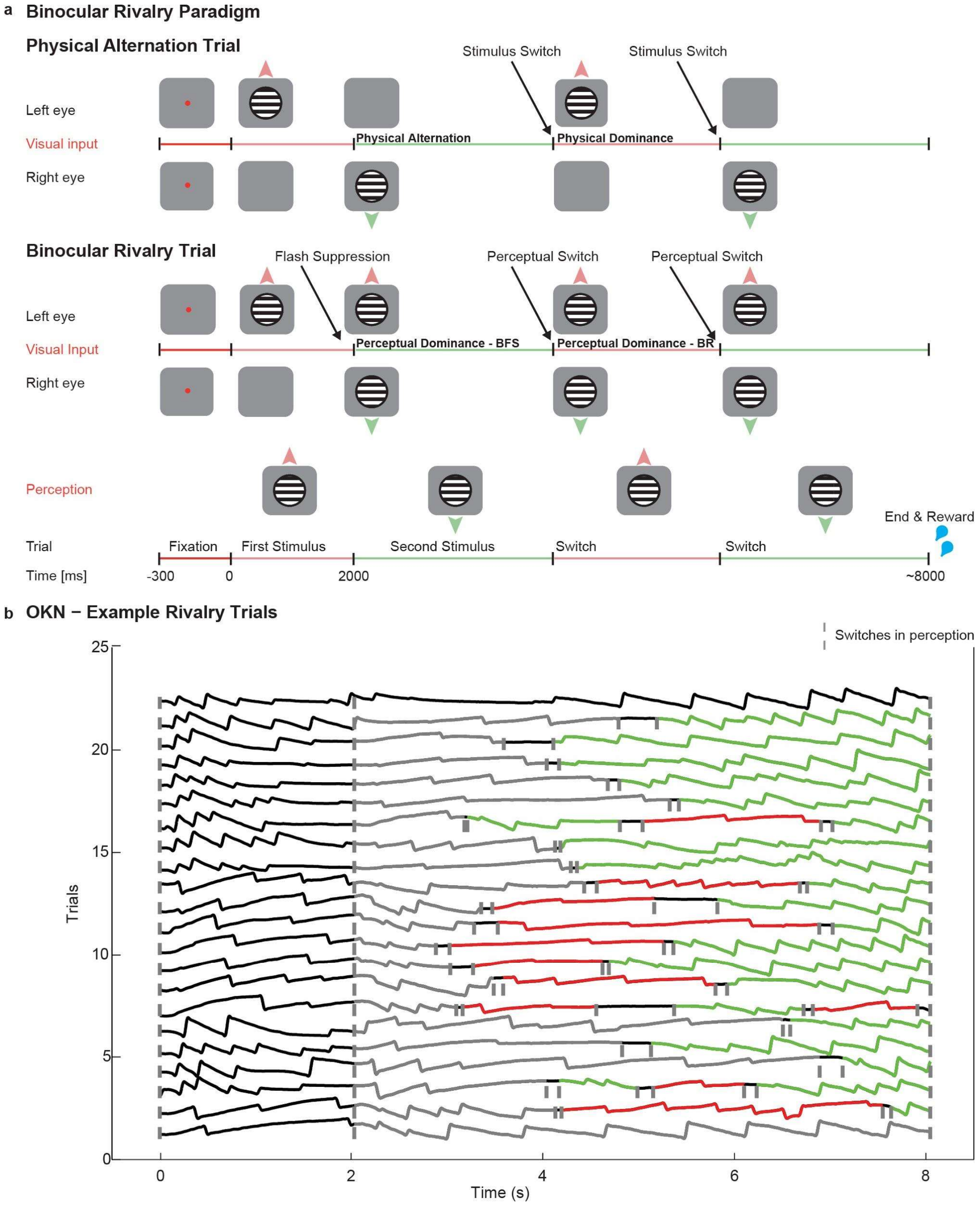

Supplementary Fig. 1: Binocular rivalry paradigm and behavior (OKN and perceptual dominance). a, The BR paradigm consisted of two trial types: physical alternation (PA) and binocular rivalry (BR) trials. Both trial types started with the presentation of a fixation spot, cueing the animal to initiate fixation. After the animal's gaze had been within the fixation window for 300 milliseconds, a drifting sinusoidal grating was monocularly presented. After 1 or 2 seconds, the first stimulus was removed and a second grating drifting in the opposite direction was presented to the contralateral eye during PA trials. During BR trials, the second stimulus was added to the contralateral eye without removing the first stimulus, inducing perceptual suppression of the first stimulus (Binocular Flash Suppression - BFS). After this period, visual input alternated between upward and downward drifting gratings during PA trials (Stimulus Switch). During BR trials, animal's perception randomly switched between the discordant visual stimuli (Perceptual Switch). Note that the perceived direction displayed in the bottom row is the same, even though the underlying visual input is monocular and unitary in PA, while it is dichoptic and ambiguous during BR. b, OKN elicited during example BR trials from one recording session. Gray vertical dashed line denotes the beginning of the flash suppression phase. Subsequent dominance phases are color coded and their beginning and end are marked with shorter grey dashed lines. Note that on the last example trial, the flash suppression resulted in prolonged continuous suppression of the previously presented stimulus, while on the first trial, flash suppression was not effective and the initially presented stimulus remained dominant.

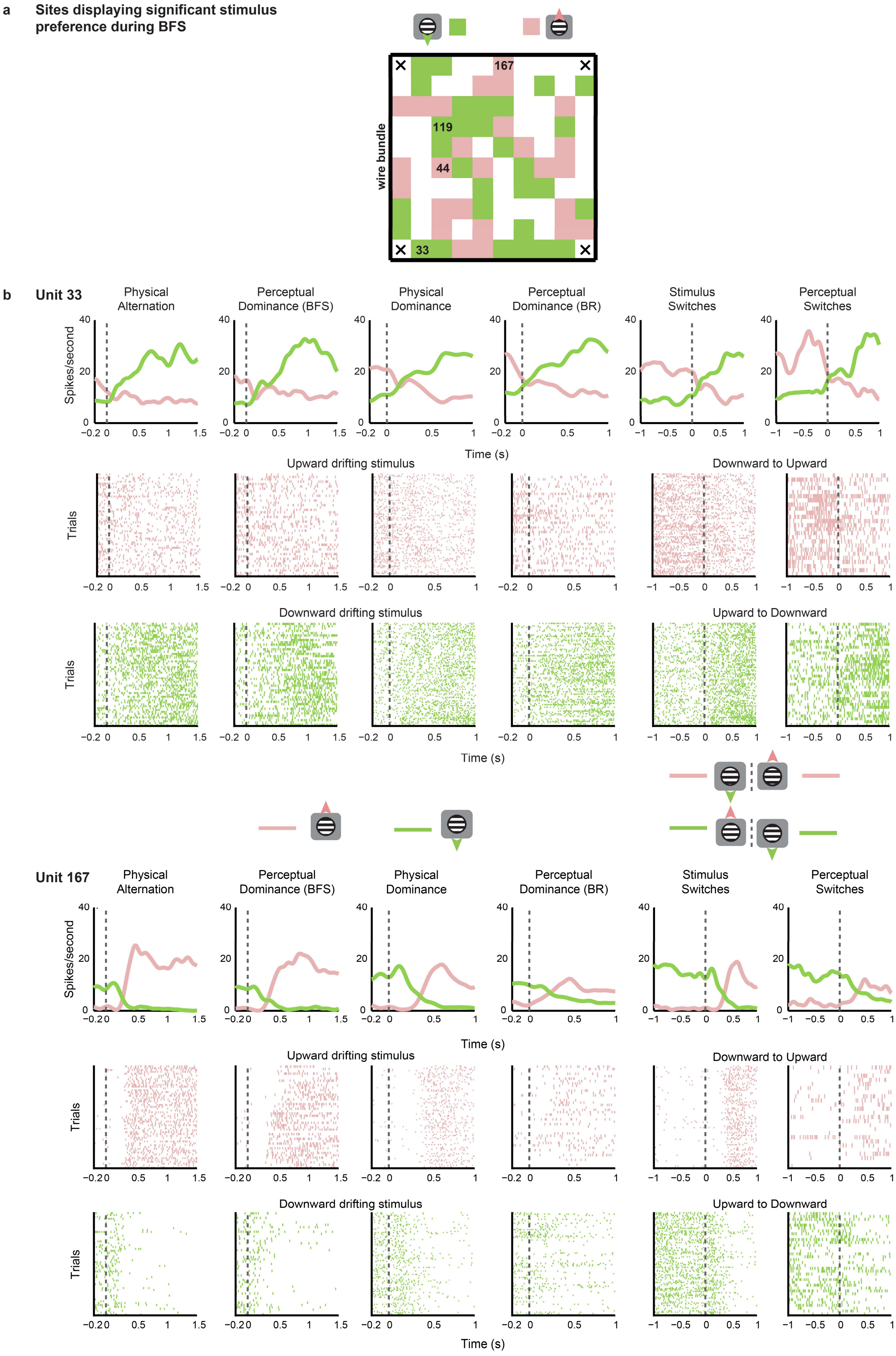
a
Sites displaying significant stimulus preference during BFS
167
119
44
33
wire bundle
b
Unit 33
Physical Alternation
Perceptual Dominance (BFS)
Physical Dominance
Perceptual Dominance (BR)
Stimulus Switches
Perceptual Switches
Spikes/second
Time (s)
Upward drifting stimulus
Downward to Upward
Trials
Downward drifting stimulus
Upward to Downward
Unit 167

Supplementary Fig. 2: Preference of sites during BFS and example unit activity. a, Sites which displayed significant stimulus preference estimated from the spiking activity elicited during the flash suppression phase of the BR trials during one recording session are projected back on the array for an example dataset. The numbers denote the location at which the units, whose activity is displayed in Fig. 1b, were recorded during a session from one animal. Green and pink pixels reflect sites, where the spiking activity (valid spiking activity recorded from a given electrode), responded more to downward or upward drifting gratings respectively. b, Spike density functions and raster plots, for the units displayed in Fig. 1e. Unit 33 displayed stronger activity in response to grating drifting down, while Unit 167 fired more, when a grating drifting up was presented in PA or perceived in BR. Stronger activity of units is evident also in the spike rasters. With respect to the first four columns of spike rasters: displayed in pink are responses related to grating drifting upwards, while in green is spiking related to the stimulus drifting down. The last two columns display spiking activity as pink rasters for a down to up switch, while in green for an up to down switch.

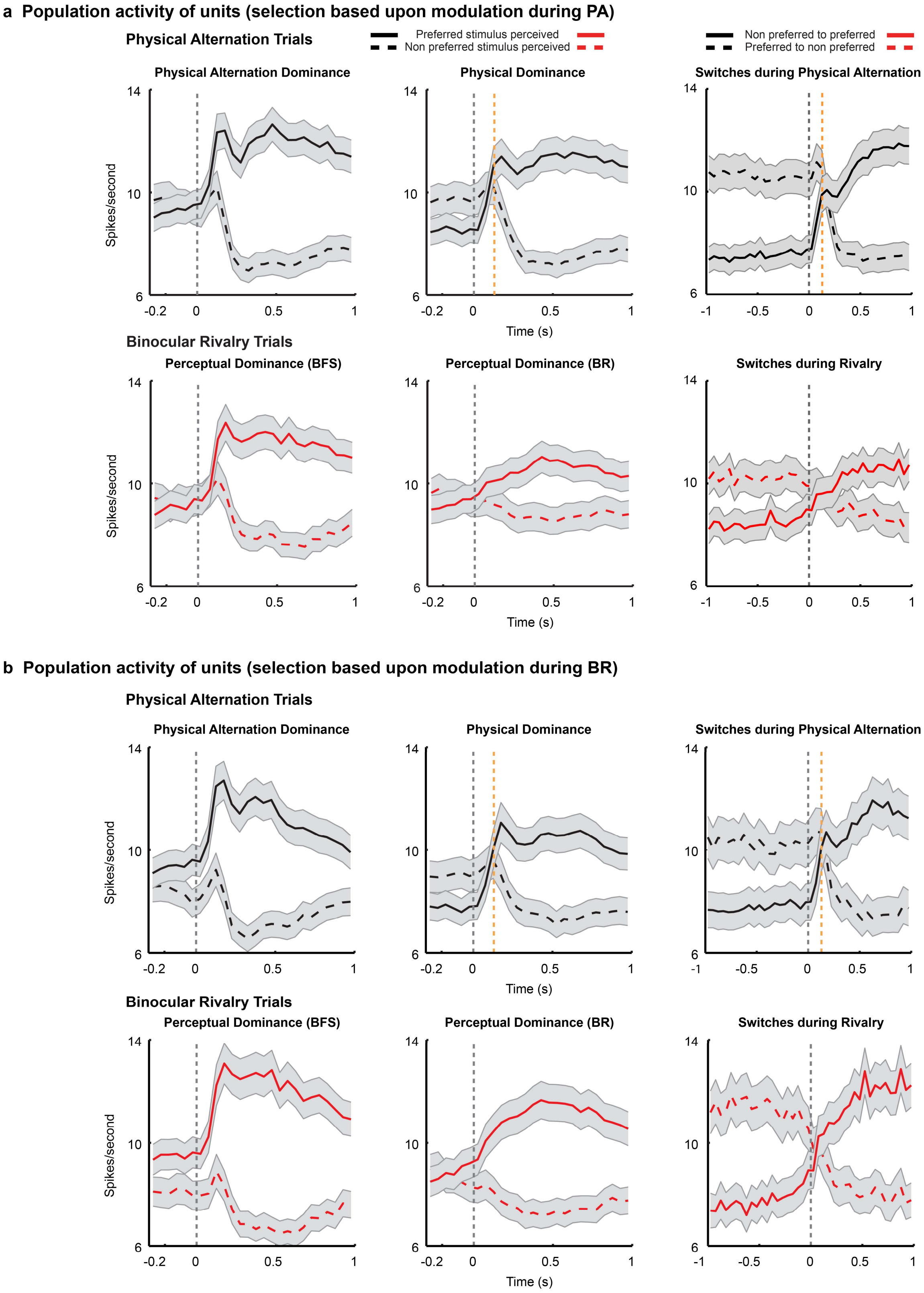

a Population activity of units (selection based upon modulation during PA)
Physical Alternation Trials
Preferred stimulus perceived
Non preferred stimulus perceived
Non preferred to preferred
Preferred to non preferred
Physical Alternation Dominance
Physical Dominance
Switches during Physical Alternation
Spikes/second
Time (s)
Binocular Rivalry Trials
Perceptual Dominance (BFS)
Perceptual Dominance (BR)
Switches during Rivalry
b Population activity of units (selection based upon modulation during BR)
Physical Alternation Trials
Physical Alternation Dominance
Physical Dominance
Switches during Physical Alternation
Binocular Rivalry Trials
Perceptual Dominance (BFS)
Perceptual Dominance (BR)
Switches during Rivalry

Supplementary Fig. 3: Population activity of units significantly modulated during a, PA or b, BR. a, Similar to Fig. 2b, the mean population spiking activity during PA (in black) and BR trials (in red) is presented across the various temporal phases of the paradigm (flash suppression, perceptual dominance and switches) averaged across all units significantly modulated during PA trials. For switches, selectivity was estimated both before and after the stimulus change. Units, which were significantly modulated either before or after a stimulus switch and preferred the same visual stimulus both before and after the stimulus switch during PA trials were used. b, Same as in a, but the population activity was computed using units that were significantly modulated during BR trials. The orange dashed line indicates the average delay of the OKN derived transition relative to the physical stimulus transition during PA trials (129.4 ± 36.6 ms). In all figure panels, the shaded regions depict standard error of the mean. Source data are provided as a Source Data file.

a Sensory versus Perceptual modulation of spiking activity - [d']

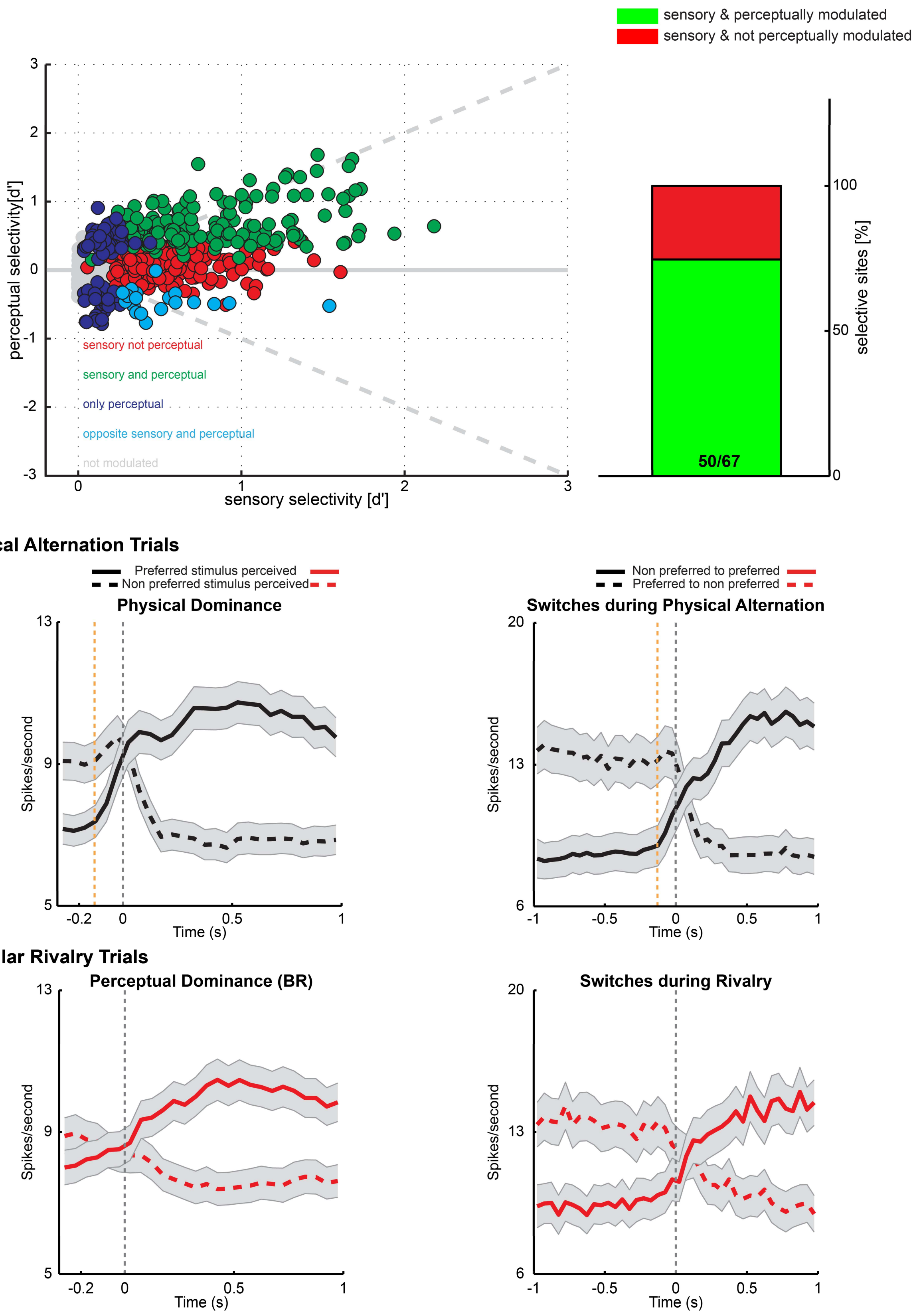

Supplementary Fig. 4: Sensory (PA) versus perceptual (BR) modulation of spiking activity - d' and population activity. Similar to Fig. 2, these plots display the results obtained when the spiking activity during PA trials was aligned to the change in OKN (see methods). a, Scatter plot of sensory versus perceptual preference index (d′) for all recorded units is displayed. Each dot denotes a unit. Units showing no significant modulation in PA or BR trials are displayed in grey while those with significant modulation during both conditions are colored green. In red are units which display significant preference only during PA trials. Units displaying significant modulation only during BR trials are displayed in blue, while in cyan are units which fired more when their preferred stimulus was perceptually suppressed. The proportion of PA modulated units, which are also significantly modulated during BR increases as a function of the strength of sensory selectivity (d′). The right column displays the proportion of PA modulated units with a d′ greater than 1, which were also significantly modulated during perceptual dominance phase in BR trials and preferred the same stimulus across conditions (green). b, Mean population spiking activity during PA (black curves) and BR trials (red curves). Similar to Fig. 2, population activity was computed by averaging across all units which were significantly modulated during PA or BR trials and preferred the same stimulus. Shaded regions depict standard error of the mean. The orange dashed line indicates the average delay of the OKN derived transition relative to the physical stimulus transition during PA trials (-129.4 ± 36.6 ms). The population activity was remarkably similar across the two trial types indicating robust perceptual modulation of neural activity in the vlPFC. Source data are provided as a Source Data file.

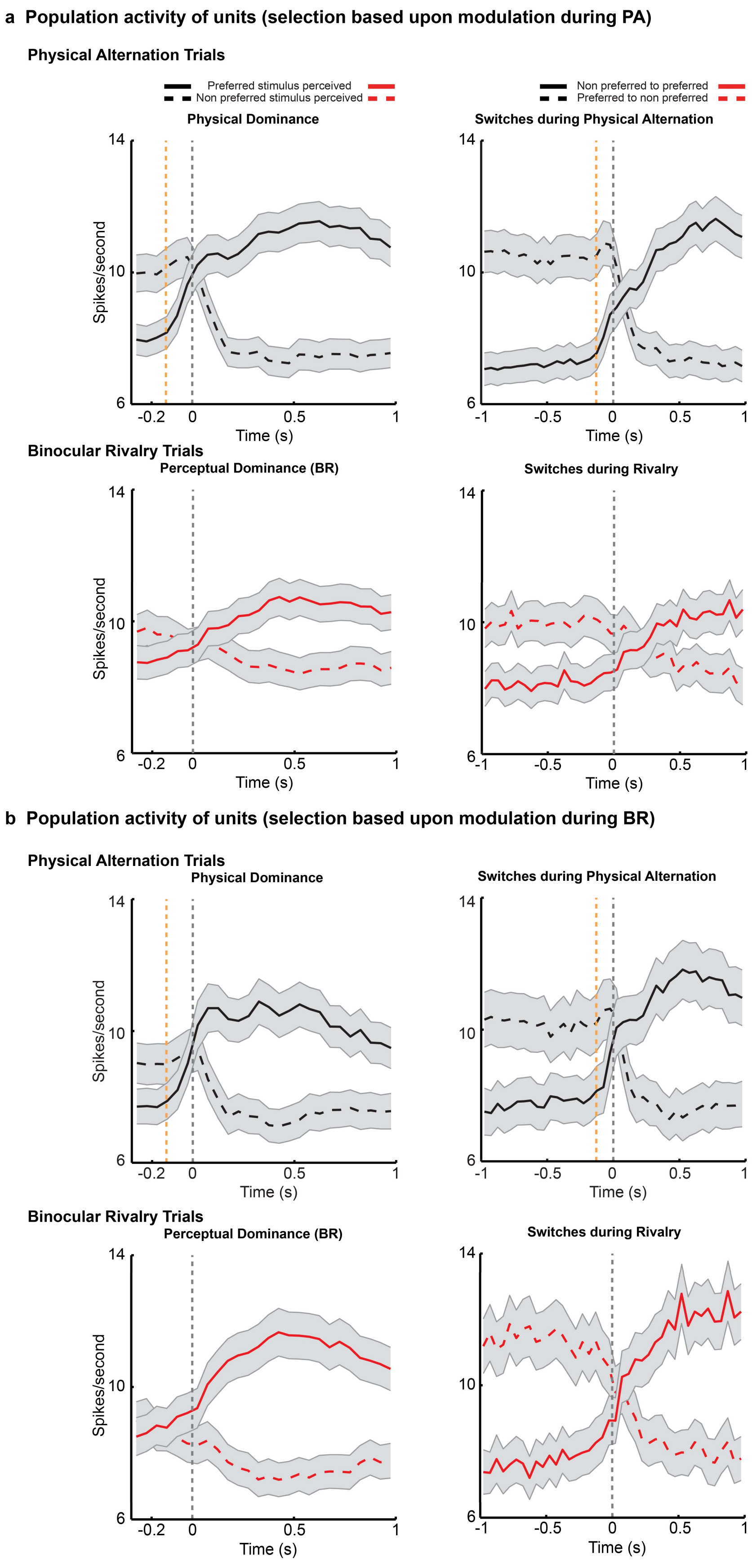

a Population activity of units (selection based upon modulation during PA)
Physical Alternation Trials
Preferred stimulus perceived
Non preferred stimulus perceived
Physical Dominance
Non preferred to preferred
Preferred to non preferred
Switches during Physical Alternation
Spikes/second
Time (s)
Binocular Rivalry Trials
Perceptual Dominance (BR)
Switches during Rivalry
b Population activity of units (selection based upon modulation during BR)
Physical Alternation Trials
Physical Dominance
Switches during Physical Alternation
Binocular Rivalry Trials
Perceptual Dominance (BR)
Switches during Rivalry

Supplementary Fig. 5: Population activity of units significantly modulated during a, PA or b, BR. a, Similar to Supplementary Fig. 3a, results obtained when spiking activity during PA trials was aligned to the change in OKN. Presented across two columns is the mean population spiking activity during PA (black curves) and BR trials (red curves). The population activity averaged across all units, which were significantly modulated during PA trials, is plotted here during two temporal phases, namely, the perceptual dominance phase and switches. b, Same as in a, but the population activity was computed using units which were significantly modulated during BR trials. The orange dashed line indicates the average delay of the physical stimulus transition relative to the OKN derived transition during PA trials (-129.4 ± 36.6 ms). In all figure panels, the shaded regions depict standard error of the mean. Source data are provided as a Source Data file.

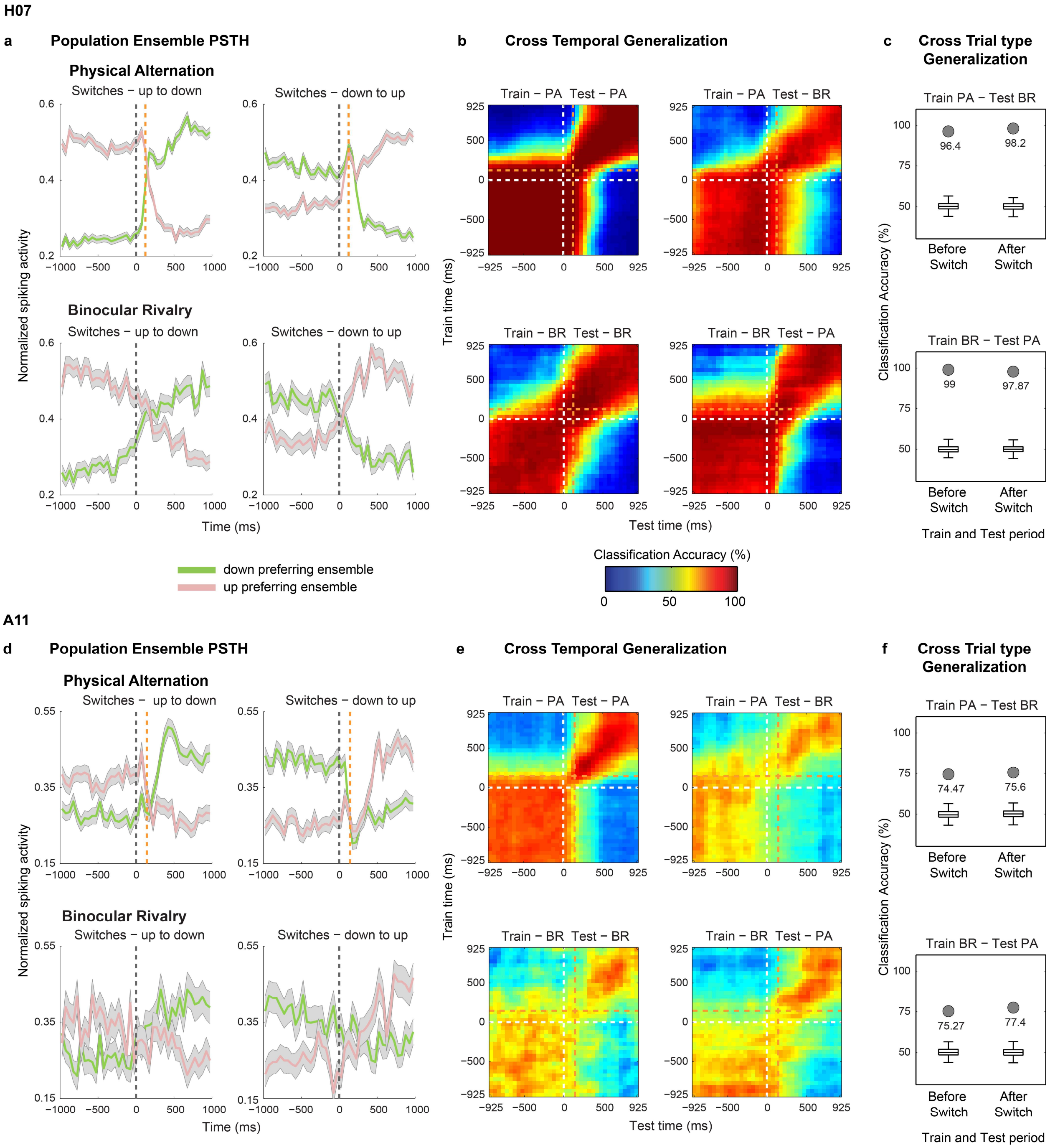

Supplementary Fig. 6: Decoding the contents of conscious perception from simultaneously recorded prefrontal ensembles in individual animals. Similar to Fig. 3a, b and c, the results obtained with the multivariate pattern analysis from individual monkeys are displayed. a, b and c display results for H07, and figures presented in d, e and f correspond to A11. Both stimulus and

perceptual contents could be successfully decoded from units recorded within an individual animal. a, d is the normalized spiking activity of neuronal ensembles (see methods) during the two different switch types. Green and pink curves denote the activity of downward and upward motion preferring ensemble respectively. Data are presented as mean and shaded regions depict standard error of the mean. b, e Cross temporal decoding and generalization within and across the two trial types. c, f A cross trial type generalization was carried out over a single temporal window of 400 ms before and after a switch. Significant generalization accuracy (permutation test, one-sided, estimated p-value: $p = 0.00199$) was obtained, when comparing it with accuracy obtained from data with shuffled labels ($n = 500$ runs, summarized as a box plots (for box plot description, see statistical information, methods)). The presented results were computed with data from single animals. In a, b, d and e, the orange dashed line indicates the average delay of the OKN derived transition relative to the physical stimulus transition during PA trials for individual monkeys (Mean ± standard deviation: 123.17 ± 34.24 ms for H07 and 144.01 ± 37.80 ms for A11). Source data are provided as a Source Data file.

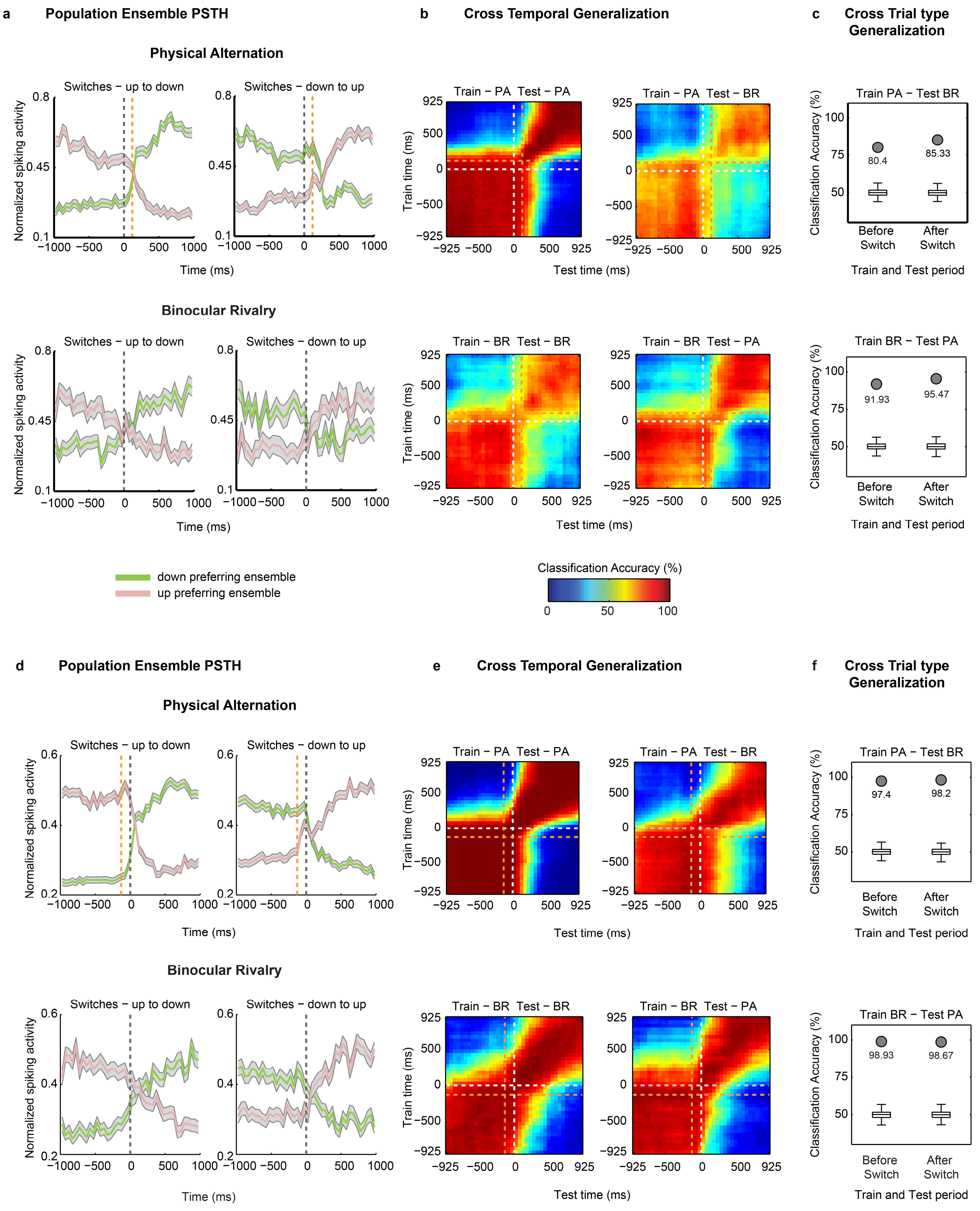
a
Population Ensemble PSTH
Physical Alternation
Switches − up to down
Switches − down to up
Normalized spiking activity
Time (ms)
Binocular Rivalry
b
Cross Temporal Generalization
Train − PA Test − PA
Train − PA Test − BR
Train − BR Test − BR
Train − BR Test − PA
Train time (ms)
Test time (ms)
c
Cross Trial type Generalization
Train PA − Test BR
Train BR − Test PA
Classification Accuracy (%)
80.4
85.33
91.93
95.47
Before Switch
After Switch
Train and Test period
down preferring ensemble
up preferring ensemble
Classification Accuracy (%)
d
Population Ensemble PSTH
Physical Alternation
Binocular Rivalry
e
Cross Temporal Generalization
f
Cross Trial type Generalization
97.4
98.2
98.93
98.67

Supplementary Fig. 7: Decoding the contents of conscious perception from simultaneously recorded prefrontal ensembles. a, b and c: Similar to Fig. 3a, b and c, the results obtained with the multivariate pattern analysis from an individual dataset are displayed. Both stimulus and perceptual contents could be successfully decoded from simultaneously recorded units in an individual dataset. d, e and f: Similar to Fig. 3, plotted here are the results obtained with the multivariate pattern analysis, when spiking activity during PA trials was aligned to the change in OKN instead of the TTL pulse (see methods). a, d is the normalized spiking activity of neuronal ensembles (see methods) during the two different switch types. Green and pink curves denote the activity of downward and upward motion preferring ensemble respectively. Data are presented as mean and shaded regions depict standard error of the mean. b, e Cross temporal decoding and generalization within and across the two trial types. c, f A cross trial type generalization was carried out over a single temporal window of 400 ms before and after a switch. We observed significant cross trial type generalization (permutation test, one-sided, estimated p-value: $p = 0.00199$), when compared to decoding accuracy obtained from data with labels shuffled ($n = 500$, summarized with box plots (for box plot description, see statistical information, methods)). In c, presented results were computed with data from a single dataset from an individual animal. Results presented in f were computed with data from two animals pooled together. In a and b, the orange dashed line indicates the average delay of the OKN derived transition relative to the physical stimulus transition during PA trials of that single dataset (118.98 ± 30.13 ms). In d and e, the average delay computed over all data of the physical stimulus transition relative to the OKN derived transition is plotted (-129.4 ± 36.6 ms). Source data are provided as a Source Data file.

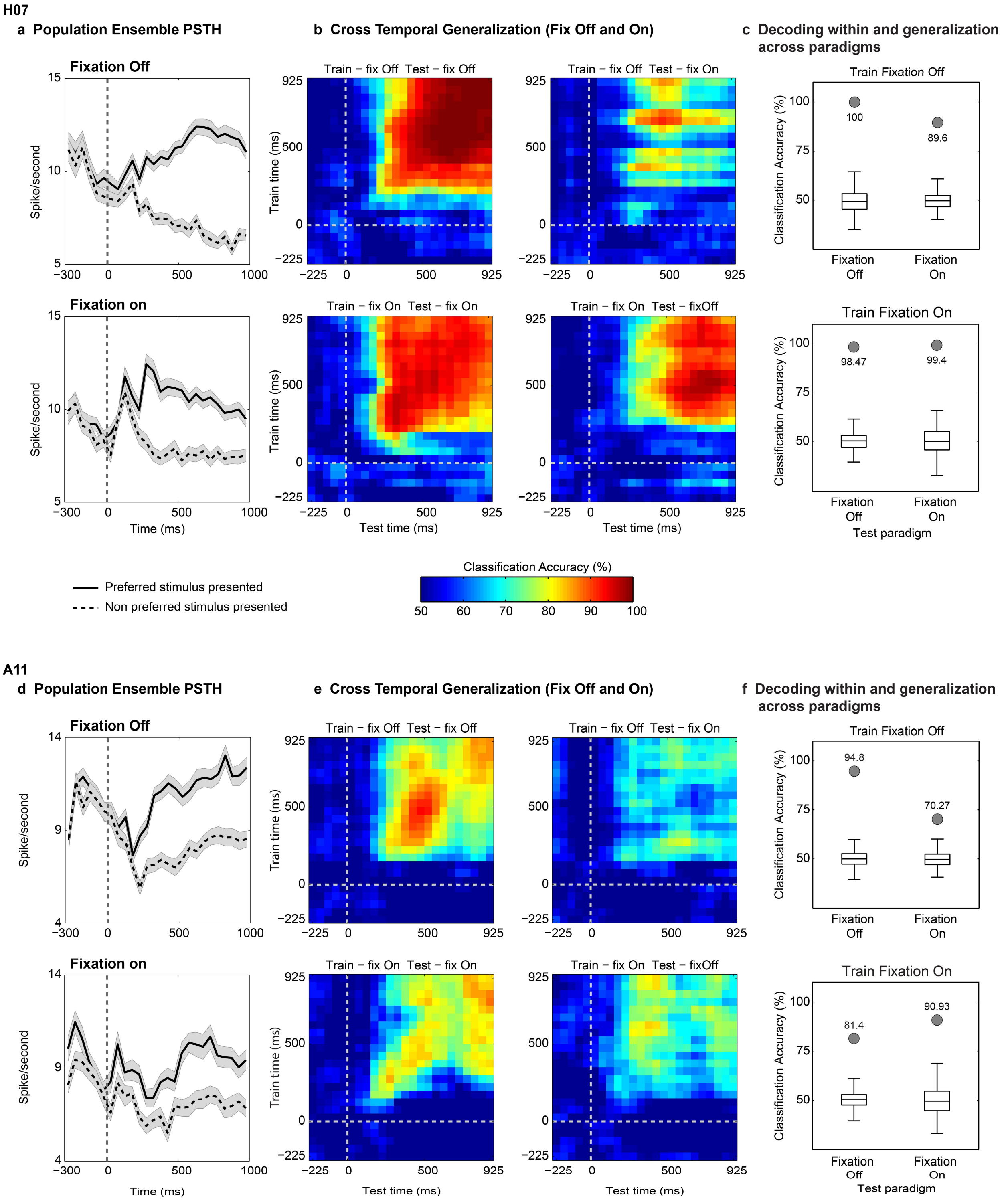

Supplementary Fig. 8: Decoding motion content (during control paradigms) from simultaneously recorded prefrontal ensembles in individual monkeys. Similar to Fig. 7, this figure summarizes the results for individual animals pertaining to the multivariate pattern analysis

assessing the invariance of the population code to motion content during the control experiments. a, d, Population spiking activity (see methods) of prefrontal ensembles is presented during Fixation off and Fixation On paradigm. This figure presents the activity of units preferring upward and downward motion combined, during the presentation of their preferred (black solid curves) or non-preferred motion direction (black dashed curves). Data are presented as mean and shaded regions depict standard error of the mean. b, e, Cross-temporal decoding of stimulus contents during the two paradigms. Similar to Fig. 7b, decoding accuracy was tested for each pair of train and test time windows. c and f, show the cross paradigm generalization of the population code, which was tested by training a classifier on activity in one paradigm and testing on the other, for a single bin of 400 ms (starting 400 ms post stimulus onset) during the presentation of the visual stimulus. Significant (permutation test, one-sided, estimated p-value: $p = 0.00199$) cross-paradigm generalization accuracy was observed across both animals, when compared with classification accuracy obtained when decoding analysis was carried out on data with shuffled labels ($n = 500$, summarized as box plots (for box plot description, see statistical information, methods)). The presented results were computed with data from single animals. Source data are provided as a Source Data file.

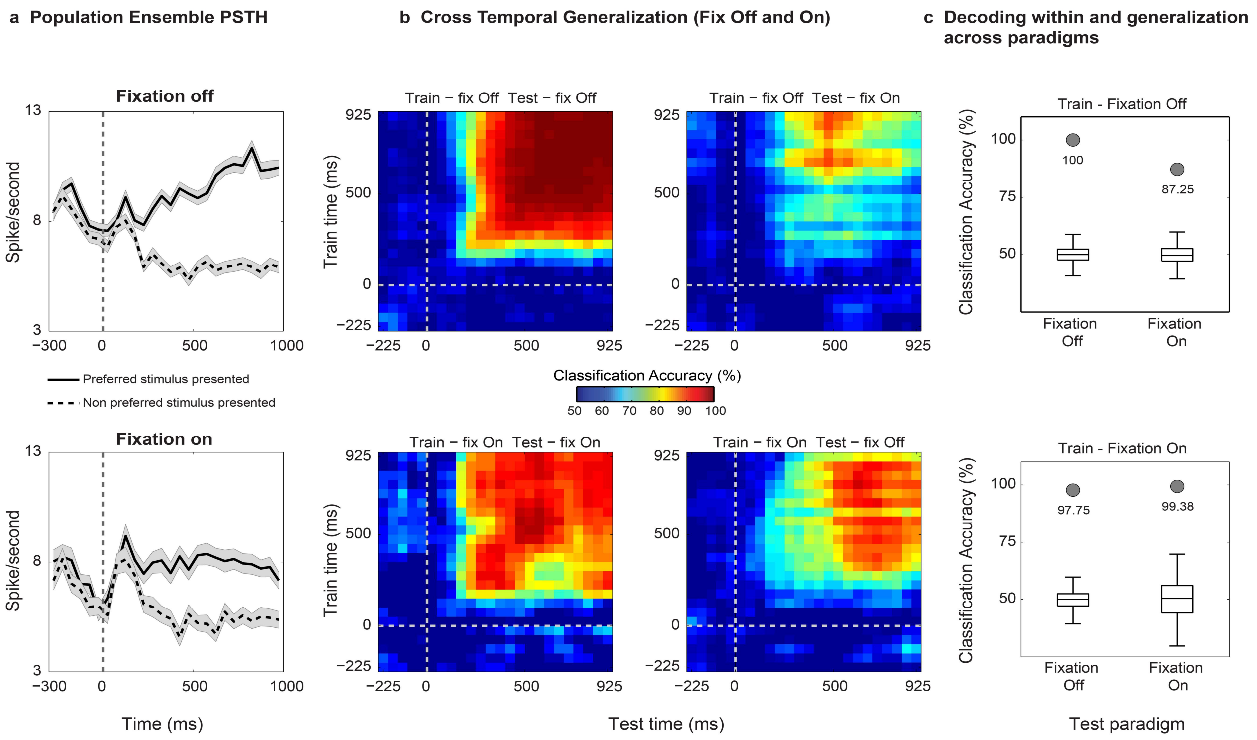

Supplementary Fig. 9: Decoding motion content (during control paradigms) from simultaneously recorded prefrontal ensembles. Similar to Fig. 7, this figure summarizes the results pertaining to the multivariate pattern analysis assessing the invariance of the population code to motion content during the control experiments. However, only a selection of trials from the Fixation On paradigm are included, where eye movements were further controlled (see methods and Figure 6). a, Population mean spiking activity (see methods) of prefrontal ensembles in response to the presentation of their preferred (black solid) and non-preferred stimulus (black dashed) during Fixation off and Fixation On paradigm. The shaded regions depict standard error of the mean. The population consisted of units, which were significantly modulated in either of the two paradigms, and preferred the same motion direction (see methods). b, Cross-temporal decoding of stimulus contents during the two paradigms. Decoding accuracy was tested for each pair of train and test time windows similar to Fig. 7b. c, The cross paradigm invariance of the population code was tested by training a classifier on activity in one paradigm and testing on the

other, for a single bin of 400 ms (starting 400 ms post stimulus onset) during the presentation of the visual stimulus. We observed significant (permutation test, one-sided, estimated p-value: $p = 0.00199$) cross-paradigm generalization accuracy, when comparing it with accuracy obtained from data with shuffled labels (summarized as box plots (for box plot description, see statistical information, methods), $n = 500$). This suggests that the underlying code is largely invariant to the presence of large OKN, and encodes stimulus motion contents. Results pertaining to decoding within the paradigm are also presented. The presented results were computed with data from two animals pooled together. Source data are provided as a Source Data file.

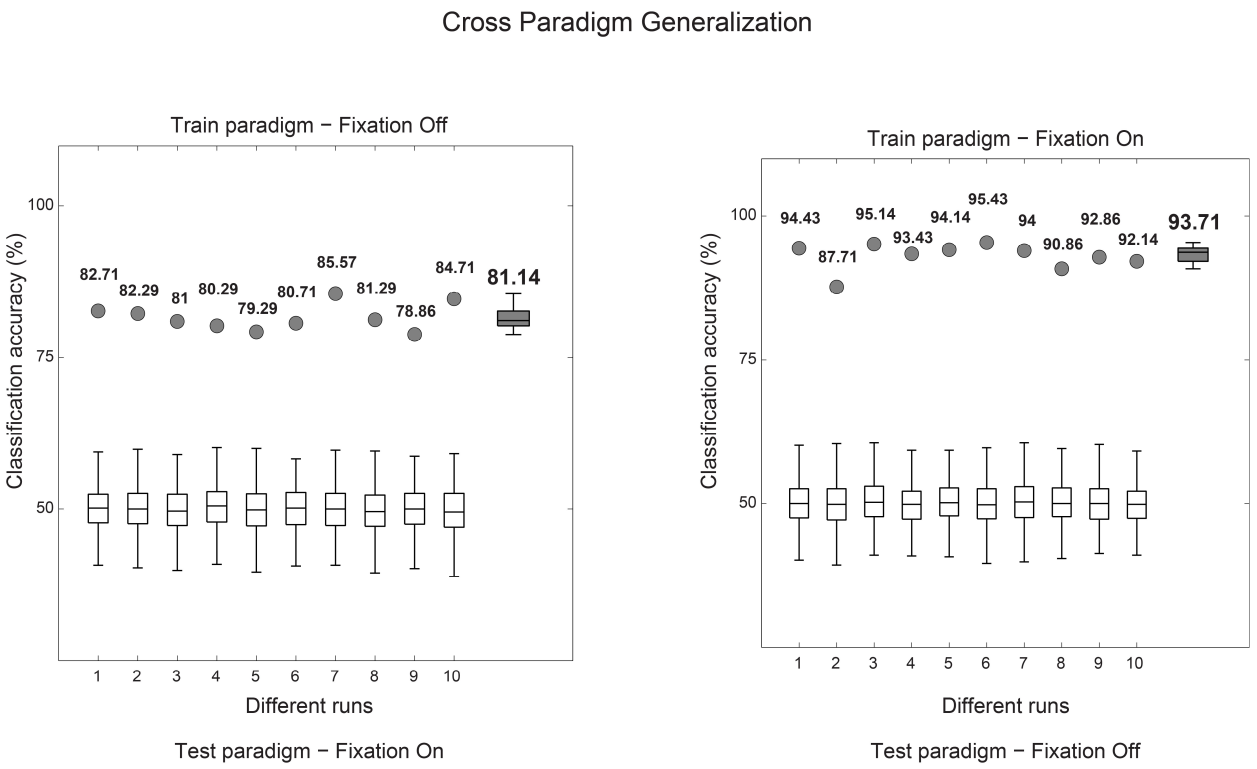

Supplementary Fig. 10: Assessment of the robustness of cross paradigm generalization to the unit selection procedure. Similar to Fig. 7c and Supplementary Fig. 9, cross paradigm invariance of the population code was tested by training a classifier on activity elicited during one paradigm and testing it on the other, for a single bin of 400 ms (starting 400 ms post stimulus onset) during visual motion presentation. However, in comparison to earlier analysis (Fig. 7c and Supplementary Fig. 9), the selection of units was performed differently and this procedure was carried out ten times (runs). For each run, the units participating in the decoding procedure were assigned by assessing their selectivity and preference computed over all trials of one condition (on which the classifier is trained) and half of the trials from the second condition. The trained classifier was then tested on the remaining half of the trials of the second condition. Filled circles denote the generalization accuracy of the classifier and the box plot (for box plot description, see statistical information, methods) on the right is estimated from all the individual values resulting from single runs. Box plots (for box plot description, see statistical information, methods) around the 50%

mark depict the distribution of the classification results ($n = 500$) obtained with shuffled labels. On each run, significant (permutation test, one-sided, estimated p-value: $p = 0.00199$) cross-task generalization accuracy was observed. These results thus confirm that the underlying population code encodes stimulus motion contents and is largely invariant to the presence of large OKN. The results presented in this figure were computed with data from two animals pooled together. Source data are provided as a Source Data file.

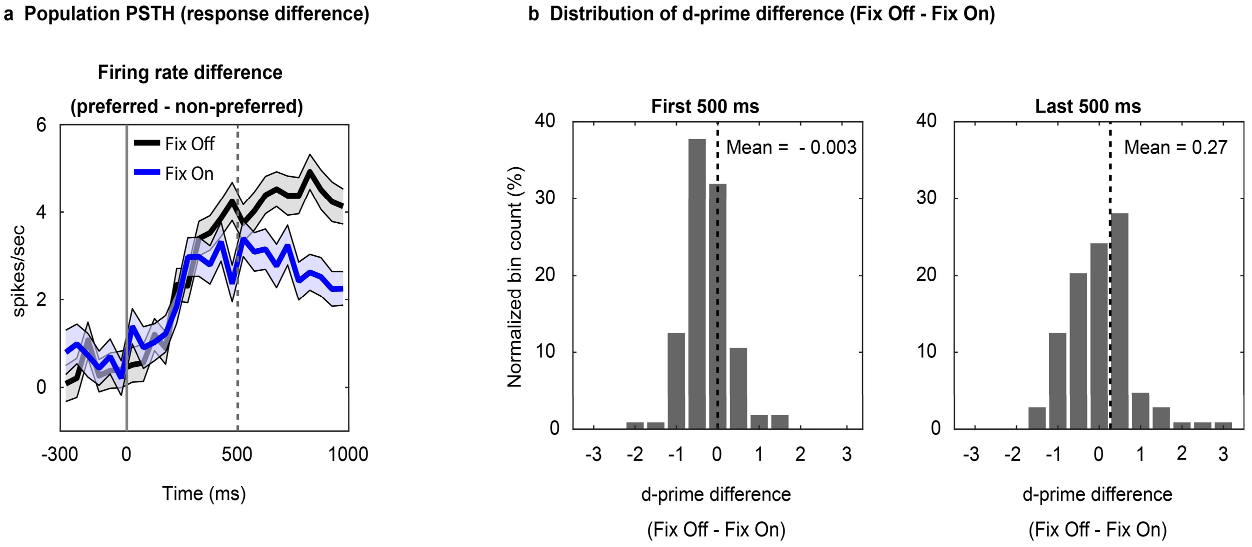

Supplementary Fig. 11: Comparison of the strength of modulation across the two control paradigms. The two curves displayed in a, correspond to the two different control paradigms, Fixation Off (black) and Fixation On (blue). Each curve is computed by averaging across units, the difference in their responses to their preferred and non-preferred stimulus in the given control paradigm. Data are presented as mean and shaded regions depict standard error of the mean. These two curves are visually similar in the first half of the trial, while in the second half, the response difference is stronger during fixation Off. b presents a quantification of this difference in response modulation by computing the difference in the d-primes of units across the two paradigms (fixation Off – fixation On) in the first and second half of the trial. While the distribution and the mean (-0.003 ± 0.05) of this difference is centered around zero for the first 500 ms (one sample t-test, one-sided, $p = 0.9552$), the mean is significantly different from zero (0.27 ± 0.07) for the last 500 ms (one sample t-test, one-sided, $p = 4.85*10^{-4}$). Source data are provided as a Source Data file.

Supplementary Table 1

Comparison of the present work with previous approaches investigating prefrontal spiking activity related to conscious perception

Electrophysiology studies investigating PFC's role in conscious visual perception		Thompson et al (1999)[1]	Libedinsky et al (2011)[2]	Panagiotaropoulos et al (2012)[3]	Gelbard-Sagiv et al (2018)[4]	van Vugt et al (2018)[5]	Present study
Region in the frontal lobe investigated		FEF	FEF	LPFC	ACC, pre-SMA	dlPFC	vlPFC
Model System		Macaques	Macaques	Macaques	Human patients	Macaques	Macaques
Conflating variables	Control for motor reports by using a no-report paradigm	✗	✗	✓	✗	✗	✓
	Control for exogenous stimulus changes, which conflate activity by inducing a change in the feedforward drive	NA	✓	✗	✓**	NA	✓**
	Control for predictability in the temporal structure of perceptual changes	✗*	✓	✗	✓**	✗***	✓**
Variables investigated	Correlating neural activity with perceptual presence and absence of visual stimulus	✓	✓	✓	✓	✓	✓
	Investigating encoding of visual features (e.g., visual motion or objects) in the neural activity during conscious perception and perceptual suppression	✗[#]	✗[#]	✓	✓	✗[#]	✓
	Investigating neural activity correlated with internally driven changes in conscious perception without changes in the external input	✗	✓	✗	✓	✗	✓
	Decoding conscious content from the activity of simultaneously recorded unit populations	✗	✗	✗	✗	✗	✓

* - Visual target presented after the monkey fixated for 500-700 ms.

** - Binocular rivalry entails stochastic (and therefore temporally unpredictable) and internally driven changes in conscious perception without a change in the sensory signal.

*** - Visual stimulus was presented after 300-500 ms of fixation.

# - The target stimuli, used in the different studies were a dim blue square[1], a yellow circle[2] or a low contrast circular stimulus[5].

Acronyms: NA – not applicable, FEF – Frontal Eye Fields, ACC – anterior cingulate cortex, pre-SMA – pre-supplementary motor area, LPFC – lateral prefrontal cortex encompassing both dorsal and ventrolateral PFC, dlPFC – dorsolateral prefrontal cortex, vlPFC – ventrolateral prefrontal cortex.

Supplementary Table 2

Number and proportion of significantly modulated units during the BR paradigm

**Trial Type**	**Physical Alternation**		**Binocular Rivalry**	
**Temporal Phase**	**Physical Alternation**	**Sensory Dominance**	**BFS Dominance**	**BR dominance**
**N of significantly modulated units**	**342**	**364**	**264**	**247**
**N and % of significantly modulated units* in PA, with similar average stimulus preference in BR**	**288/342 84.21%**	**277/364 76.09%**		
**N and % of significantly modulated units* in BR, with similar stimulus preference in PA**			**229/264 86.74%**	**199/247 80.56%**
**N of significantly modulated units in PA with a d'>1**	**80**	**46**		
**N and % of significantly modulated units* in PA (d'>1), with similar preference and significantly modulated in BR (ALL)**	**72/80 90%**	**40/46 86.96%**		

*significantly modulated here in a particular condition refers to units displaying significantly stronger activity to one of the two directions of motion assessed with a Wilcoxon rank sum test at an alpha value of 0.05.

Supplementary Table 3

Number and proportion of significantly modulated units during the BR paradigm (Physical alternation trials aligned to the change in the OKN direction)

Trial Type	Physical Alternation	Binocular Rivalry
Temporal Phase	Sensory Dominance	BR dominance
N of significantly modulated units	410	247
N and % of significantly modulated units* in PA, with similar average stimulus preference in BR	325/410<br>79.27%	
N and % of significantly modulated units*in BR, with similar stimulus preference in PA		199/247<br>80.56%
N of significantly modulated units in PA with a d'>1	67	
N and % of significantly modulated units* in PA (d'>1), with similar preference and significantly modulated in BR (ALL)	50/67<br>74.63%	

SUPPLEMENTARY REFERENCES

1. **Thompson, K. G. & Schall, J. D. The detection of visual signals by macaque frontal eye field during masking. *Nat. Neurosci.* 2, 283–288 (1999).**
2. **Libedinsky, C. & Livingstone, M. Role of prefrontal cortex in conscious visual perception. *J. Neurosci.* 31, 64–69 (2011).**
3. **Panagiotaropoulos, T. I., Deco, G., Kapoor, V. & Logothetis, N. K. Neuronal discharges and gamma oscillations explicitly reflect visual consciousness in the lateral prefrontal cortex. *Neuron* 74, 924–935 (2012).**
4. **Gelbard-Sagiv, H., Mudrik, L., Hill, M. R., Koch, C. & Fried, I. Human single neuron activity precedes emergence of conscious perception. *Nat. Commun.* 9, 2057 (2018).**
5. **van Vugt, B. *et al.* The threshold for conscious report: Signal loss and response bias in visual and frontal cortex. *Science* 360, 537–542 (2018).**
