# Supplementary material for: Decoding internally generated transitions of conscious contents in the prefrontal cortex without subjective reports
Source: Nat Commun. 2022 Mar 22;13:1535. doi: 10.1038/s41467-022-28897-2 (PMC8940963; doi:10.1038/s41467-022-28897-2)
Supplement: Supplementary file 2 — Reporting Summary [file 41467_2022_28897_MOESM2_ESM.pdf]

nature research

Corresponding author(s): Vishal Kapoor, Theofanis Panagiotaropoulos

Last updated by author(s): Dec 24, 2021

Reporting Summary

Nature Research wishes to improve the reproducibility of the work that we publish. This form provides structure for consistency and transparency in reporting. For further information on Nature Research policies, see our Editorial Policies and the Editorial Policy Checklist.

Statistics

For all statistical analyses, confirm that the following items are present in the figure legend, table legend, main text, or Methods section.

n/a	Confirmed	
☐	☒	The exact sample size (*n*) for each experimental group/condition, given as a discrete number and unit of measurement
☒	☐	A statement on whether measurements were taken from distinct samples or whether the same sample was measured repeatedly
☐	☒	The statistical test(s) used AND whether they are one- or two-sided<br>*Only common tests should be described solely by name; describe more complex techniques in the Methods section.*
☒	☐	A description of all covariates tested
☒	☐	A description of any assumptions or corrections, such as tests of normality and adjustment for multiple comparisons
☐	☒	A full description of the statistical parameters including central tendency (e.g. means) or other basic estimates (e.g. regression coefficient) AND variation (e.g. standard deviation) or associated estimates of uncertainty (e.g. confidence intervals)
☐	☒	For null hypothesis testing, the test statistic (e.g. *F*, *t*, *r*) with confidence intervals, effect sizes, degrees of freedom and *P* value noted<br>*Give P values as exact values whenever suitable.*
☒	☐	For Bayesian analysis, information on the choice of priors and Markov chain Monte Carlo settings
☒	☐	For hierarchical and complex designs, identification of the appropriate level for tests and full reporting of outcomes
☒	☐	Estimates of effect sizes (e.g. Cohen's *d*, Pearson's *r*), indicating how they were calculated

Our web collection on statistics for biologists contains articles on many of the points above.

Software and code

Policy information about availability of computer code

Data collection	A stereoscope was used for presentation of visual stimuli. They were displayed at a resolution of 1,280×1,024 on the monitors (running at a 60 Hz refresh rate) using a dedicated graphics workstation (TDZ 2000; Integraph Systems, Huntsvilli, AL, USA). Visual stimulus presentation was carried out using openGL (version 1.2) and controlled on a windows machine via Tcl/Tk (version 8.0). Overall control of the experiment was realized via a real time operating system, called QNX (version 4.25).<br>Blackrock data acquisition system (Blackrock Microsystems) was used to acquire and store broadband neural signal recorded with UTAH arrays.
Data analysis	We used routines implemented as part of the neural decoding toolbox for carrying out the various decoding analysis reported within the paper. These are available online (http://www.readout.info/). This along with other code written in MATLAB R2013b (MathWorks, Natick, MA, USA) was used for data and statistical analysis.

For manuscripts utilizing custom algorithms or software that are central to the research but not yet described in published literature, software must be made available to editors and reviewers. We strongly encourage code deposition in a community repository (e.g. GitHub). See the Nature Research guidelines for submitting code & software for further information.

Data

Policy information about availability of data

All manuscripts must include a data availability statement. This statement should provide the following information, where applicable:

- Accession codes, unique identifiers, or web links for publicly available datasets
- A list of figures that have associated raw data
- A description of any restrictions on data availability

Source data are provided with this paper. Rest of the data that support the findings of this study are available from the corresponding author upon reasonable request.

Field-specific reporting

Please select the one below that is the best fit for your research. If you are not sure, read the appropriate sections before making your selection.

☒ Life sciences ☐ Behavioural & social sciences ☐ Ecological, evolutionary & environmental sciences

For a reference copy of the document with all sections, see nature.com/documents/nr-reporting-summary-flat.pdf

Life sciences study design

All studies must disclose on these points even when the disclosure is negative.

Sample size	For the present study, we used 2 rhesus macaques. This is typically the number and convention for systems neuroscience studies and considered sufficient for generalization of results.
Data exclusions	Various analysis related to the binocular rivalry paradigm included all units recorded across the given datasets (recording sessions). For the decoding analysis pertaining to control experiments, we utilized a set of selective units (selectivity criterion is detailed in the manuscript). We note that all units underwent the selectivity analysis and we further assessed the robustness of this unit selection procedure.
Replication	We found units modulated by the presented motion direction in the prefrontal cortex of both animals. As detailed in the manuscript, we obtained a similar overall pattern of results pertaining to the decoding analysis, when we analyzed the data from the two individual animals separately.
Randomization	The present study does not compare across treatment conditions. Therefore, allocation of organisms to different groups is not applicable to this study, since we are not comparing across treatment conditions. Therefore, organisms were not randomized for the experiments.
Blinding	Given that the experiments investigated the healthy brain, no blinding was needed. We did not have separate experimental groups and therefore blinding was not needed.

Reporting for specific materials, systems and methods

We require information from authors about some types of materials, experimental systems and methods used in many studies. Here, indicate whether each material, system or method listed is relevant to your study. If you are not sure if a list item applies to your research, read the appropriate section before selecting a response.

Materials & experimental systems

n/a	Involved in the study	
☒	☐	Antibodies
☒	☐	Eukaryotic cell lines
☒	☐	Palaeontology and archaeology
☐	☒	Animals and other organisms
☒	☐	Human research participants
☒	☐	Clinical data
☒	☐	Dual use research of concern

Methods

n/a	Involved in the study	
☒	☐	ChIP-seq
☒	☐	Flow cytometry
☒	☐	MRI-based neuroimaging

Animals and other organisms

Policy information about studies involving animals; ARRIVE guidelines recommended for reporting animal research

Laboratory animals	Two healthy male rhesus monkeys (Macaca mullatta), aged ~12 and ~15 years participated in electrophysiological recordings.
Wild animals	The study did not involve wild animals.

Field-collected samples	The study did not involve samples collected from the field.
Ethics oversight	All experiments were approved by the local authorities (Regierungspräsidium, Tuebingen, Baden-Württemberg, Germany, protocol KY6/12) and were in full compliance with the guidelines of the European community (2010/63/EU) for the care and use of laboratory animals.

Note that full information on the approval of the study protocol must also be provided in the manuscript.
